# Nano-Drugs Based on Nano Sterically Stabilized Liposomes for the Treatment of Inflammatory Neurodegenerative Diseases

**DOI:** 10.1371/journal.pone.0130442

**Published:** 2015-07-06

**Authors:** Keren Turjeman, Yaelle Bavli, Pablo Kizelsztein, Yaelle Schilt, Nahum Allon, Tamar Blumenfeld Katzir, Efrat Sasson, Uri Raviv, Haim Ovadia, Yechezkel Barenholz

**Affiliations:** 1 Laboratory of Membrane and Liposome Research, Department of Biochemistry and Molecular Biology, Institute for Medical Research Israel-Canada (IMRIC), The Hebrew University-Hadassah Medical School, Jerusalem, Israel; 2 The Institute of Chemistry, The Hebrew University of Jerusalem, Edmond J. Safra Campus, Givat Ram, Jerusalem, Israel; 3 BioImage MRI Research & Consulting, Tel Aviv, Israel; 4 Department of Neurology, Agnes Ginges Center for Human Neurogenetics, Hadassah University Hospital, Jerusalem, Israel; Charite Universitätsmedizin Berlin, GERMANY

## Abstract

The present study shows the advantages of liposome-based nano-drugs as a novel strategy of delivering active pharmaceutical ingredients for treatment of neurodegenerative diseases that involve neuroinflammation. We used the most common animal model for multiple sclerosis (MS), mice experimental autoimmune encephalomyelitis (EAE). The main challenges to overcome are the drugs’ unfavorable pharmacokinetics and biodistribution, which result in inadequate therapeutic efficacy and in drug toxicity (due to high and repeated dosage). We designed two different liposomal nano-drugs, i.e., nano sterically stabilized liposomes (NSSL), remote loaded with: (a) a “water-soluble” amphipathic weak acid glucocorticosteroid prodrug, methylprednisolone hemisuccinate (MPS) or (b) the amphipathic weak base nitroxide, Tempamine (TMN). For the NSSL-MPS we also compared the effect of passive targeting alone and of active targeting based on short peptide fragments of ApoE or of β-amyloid. Our results clearly show that for NSSL-MPS, active targeting is not superior to passive targeting. For the NSSL-MPS and the NSSL-TMN it was demonstrated that these nano-drugs ameliorate the clinical signs and the pathology of EAE. We have further investigated the MPS nano-drug’s therapeutic efficacy and its mechanism of action in both the acute and the adoptive transfer EAE models, as well as optimizing the perfomance of the TMN nano-drug. The highly efficacious anti-inflammatory therapeutic feature of these two nano-drugs meets the criteria of disease-modifying drugs and supports further development and evaluation of these nano-drugs as potential therapeutic agents for diseases with an inflammatory component.

## Introduction

A wide range of central nervous system (CNS) disorders and especially neurodegenerative disorders are associated with neuroinflammation [1, 2], as exemplified by multiple sclerosis (MS), the most common cause of neurological disability in young adults [3, 4]. It is characterized by the infiltration of the CNS by leukocytes, leading to substantial inflammation and damage to myelin and axons, resulting in neuronal dysfunction. MS manifests itself by relapses and remissions of neurological disturbance. As a result of continuing neuroinflammation, damage to neurons and axons slowly progresses to permanent physical disability [3, 4]. There is no known cure for multiple sclerosis. Treatments attempt to return function after an attack, minimize new attacks, and prevent disability [5]. The most commonly used animal model for MS is experimental autoimmune encephalomyelitis (EAE), which resembles the inflammatory acute phase of the human disease. The onset of EAE is characterized by enhanced vascular permeability of the blood-brain barrier (BBB) and the initiation of an inflammatory response [3, 6, 7]. Further worsening of the disease eventually leads to severe morbidity and even death [8]. Such diseases are especially amenable to treatment by nano-drug delivery systems since intra-lesional injection is not feasible, and the disruption of the BBB can be used as the Achilles’ heel of the disease as it enables one to achieve passive and active accumulation of nanoparticles at the disease site. We designed two liposomal nano-drugs aimed at the systemic treatment of diseases that involve inflammation, including MS. The objective of using such nano-drugs is to achieve a high drug concentration in the inflamed target tissue (the diseased CNS) while avoiding irrelevant tissues; such treatment will be much superior to an equivalent dose given as free drug. This should allow using a lower total amount of drug and frequency of administration, thereby reducing unwanted side effects. In this study we compare two very different liposomal nano-drug formulations, both based on nano sterically stabilized liposomes (NSSL) we have described before [9–13]. The first formulation was a liposomal glucocorticosteroid (NSSL-GC), remote loaded with the commonly used methylprednisolone hemisuccinate sodium salt (NSSL-MPS), which is a water-soluble, amphipathic weak acid and a prodrug of the glucocorticoid, methylprednisolone [12]. This prodrug is converted rapidly to the active pharmaceutical ingredient (API) methylprednisolone (MP) upon hydrolysis by the ubiquitous esterases found in biological fluids and inside cells. MP has been used for the treatment of multiple sclerosis acute relapses for over 50 years. In MS, a pulse therapy with 10 mg/kg MP for 3–5 days is the standard regimen in relapse therapy [14, 15]. Despite the efficacy of GCs in the treatment of MS, such therapies are accompanied by multiple side effects [16, 17]. These side effects depend on the duration, dose, and combination with other therapeutic agents, and are expected to be reduced when the GC is administered as a liposomal drug. In addition, effective treatment with glucocorticoids requires frequent intravenous injections and hospitalization, reducing patient compliance. The dose, as well as dosing frequency, of glucocorticoids can be reduced by incorporating them in pegylated nano-liposomes, resulting in reduced systemic toxicity while maintaining the same or even improved efficacy [12, 18–20]. The goal of our study with the use of NSSL-MPS to treat EAE (as a model of MS) was to investigate in depth its mechanism of action (MoA). The second nano-drug formulation used was pegylated nano-liposomes remote loaded with Tempamine, (NSSL-TMN). TMN acts as an antioxidant and anti-inflammatory agent [11]. Brain pathology, as in the case of neuroautoimmune diseases, is worsened, not only by the infiltration of activated lymphoid cells, but also by the release of large amounts of free radicals [21]. To neutralize these free radicals there is a need for a highly potent antioxidant that can reach the diseased tissue in the brain. Many attempts that were made in the past to use natural antioxidants such as vitamin E, carotenoids, flavonoids, and SOD failed, as these agents do not reach the diseased brain at sufficient levels [21–27]. TMN, a nitroxide having potent antioxidant activities [28], was selected as the drug of choice because it is an amphipathic weak base (due to its primary amino group) with a proven ability to be efficiently remote loaded into nano-liposomes and to be released from them at the diseased tissue [13]. Piperidine nitroxides such as Tempol, Tempo, and TMN are cell-permeable, nontoxic, and nonimmunogenic stable cyclic radicals [28, 29]. Nitroxides exert their antioxidant activity through several mechanisms: SOD-mimicking, oxidation of reduced metal ions, reduction of hypervalent metals, and interruption of radical chain reactions [30, 31]. In addition, nitroxides are also anti-inflammatory drugs [11, 32, 33]. We have previously reported that systemic administration of NSSL-TMN ameliorates the “clinical” manifestations of EAE [11]. The MoA in EAE is probably related to the ability of nitroxides to trap and neutralize free radicals, and to decrease lymphocyte activation and cytokine release, resulting in amelioration of tissue damage [11]. We also demonstrated that the TMN release rate depends on the type of ammonium salt anion and on NSSL lipid composition [13]. Liposomes having the low-T_m_ “fluid” egg phosphatidylcholine (EPC) as their “liposome-forming lipid” release TMN much faster than liposomes having the high-T_m_ “solid” hydrogenated soybean phosphatidylcholine (HSPC) as the liposome-forming lipid. Although the high resistance to release is important for long shelf-life stability, it may reduce the therapeutic efficacy due to a slower release rate at 37°C. Further development of NSSL-TMN as a potential therapeutic agent for immune-related diseases (such as multiple sclerosis) is needed.

The present study is aimed to characterize the activity and MoA of two novel nano-drugs with different treatment modalities for the treatment of neurodegenerative diseases involving an inflammatory component, as exemplified by the use of an established EAE mice model.

## Materials and Methods

### Materials

#### Lipids

Highly pure phospholipids were used. Egg phosphatidylcholine, (EPC S, 99.1% pure, T_m_ -5°C, batch no. 108064-03/103); hydrogenated soybean phosphatidylcholine (HSPC, ≥98% pure, T_m_ 52.5°C, batch no. 256270-1/58); 1,2-dimyristoyl-*sn*-glycero-3-phosphocholine (DMPC, ≥99 pure, T_m_ 24°C, batch no. 562212-1/25); 1,2-dipalmitoyl-*sn*-glycero-3-phosphocholine (DPPC, ≥99 pure, T_m_ 41.4°C, batch no. 563098-1/905); and cholesterol (NF grade) were all obtained from Lipoid (Ludwigshafen, Germany). PEG-DSPE-2000 was obtained from Genzyme Pharmaceuticals (Liestal, Switzerland).

#### Drugs

MPS (SOLU-MEDROL^TM^) was obtained from Pfizer. The free radical, 2,2,6,6-tetramethylpiperidine-4-amino-1-oxyl (4-amino-Tempo, termed Tempamine and abbreviated TMN) free base, 97% pure, was purchased from Aldrich. Hydrocortisone succinate sodium salt (HC) was obtained from Sigma.

#### Other Reagents

Highly pure water was obtained using the WaterPro PS HPLC/Ultrafilter Hybrid System (Labconco, Kansas City, MO). HPLC-grade ethanol and acetonitrile were obtained from BioLab Ltd, Jerusalem, Israel. Glacial acetic acid was obtained from Frutarom, Israel. All other chemicals were obtained from Sigma. Dialysis membrane (Cellu Sep, regenerated cellulose tubular membrane) was purchased from Membrane Filtration Products, Inc. (Seguin, Texas, USA).

#### Animals

SJL/J mice were obtained from Harlan Laboratories (Jerusalem, Israel).

The animals were housed under standard conditions of 12-hour light/dark cycle at the Hebrew University Animal Facility and given food and water ad libitum. All experiments were carried out in strict accordance with protocols approved by the Animal Ethical Care Committee of The Hebrew University of Jerusalem Medical School (MD-09-11840-5). Daily observation, weighing, and scoring of mice started on Day 7 and continued until the end of the study. During periods of active paralysis, animals were separated from the non-affected animals, provided with moistened food on the cage floor, and injected subcutaneously with saline as hydration fluid. Animals were euthanized when they met the following humane endpoint criteria: loss of >20% of the initial body weight, or a >10% loss compared to the previous measured body weight, or having full clinical paralysis. Animals were sacrificed once the experiment was completed. In all cases, mice were sedated (using carbon dioxide) prior to cervical dislocation.

### Methods

#### Preparation and characterization of drug-loaded NSSL

NSSL-MPS were fabricated and characterized as described in Avnir et al. [9, 10]. Egg phosphatidylcholine (EPC)-based NSSL-TMN were prepared and characterized as described by Wasserman et al. [13] and Kizelstein et al. [11]. In short, a mixture of the desired PC (or mixture of DMPC and DPPC at 24 to 30 mole ratio), cholesterol, and 2000-PEG-DSPE (in 55:40:5 mole ratio or 24:30:41:5 mole ratio for the DMPC:DPPC-based NSSL) was dissolved in ethanol to a concentration of 60% w/v. This solution was injected into the desired concentration of 250 mM aqueous solution of calcium acetate for MPS remote loading, or ammonium sulfate for TMN remote loading in order to hydrate the lipids and form a dispersion of multilamellar liposomes, MLV, (of 10% lipid (w/v)). These MLV were then downsized by sequential extrusion steps, done above the SO-to-LD phase transition temperature, through polycarbonate filters of decreasing defined pore size, starting with 400-nm and ending with 50-nm pore-size filters under increasing nitrogen pressure (up to 200 psi), using the Northern Lipids, Inc. (Burnaby, BC, Canada) extruder device. Intraliposomal salt gradient (i.e., calcium acetate or ammonium sulfate) was created by dialyzing the NSSL against either 10% sucrose or 5% dextrose, for MPS remote loading, and 0.13 M NaCl in 0.01 M Na citrate buffer, for TMN remote loading in a few steps overnight at 4°C. The NSSL formulated had a narrow size distribution (average d. nm ± STD) of 74.2 ± 3.47 for NSSL-MPS and 74.3 ± 4.6 for NSSL-TMN). The amount of entrapped TMN in liposomes was determined using Electron-Spin Resonance Spectroscopy (ESR) [13]. A Magnettech ESR Miniscope MS 100 spectrometer (Berlin, Germany), equipped with a Microwave X-band Bridge was used to detect TMN concentration. The spectrometer operates at 9.3−9.55 GHz and 20 mW microwave power. Samples were examined inside a glass capillary. Measurements were conducted at room temperature with the following typical parameters: B0 field: 3360 G; sweep width: 100 G; scan time: 30 s; number of scans: 3; modulation amplitude: 1.2 G; receiver gain: 10–50. Quantification was carried out as the sum of total amplitude on first derivation of ESR signal, and outcomes are expressed as the total amplitude in arbitrary units. Kinetics of TMN release from NSSL (EPC- or DMPC:DPPC-based NSSL) at 5, 25, and 37°C was followed for 133 days and determined using the ESR method as described above. The concentrations of total MPS and intraliposomal MPS, (obtained after treatment with Dowex 2X-800 anion exchanger at pH 5, which binds all free drug) and MPS hydrolysis products were quantified using the previously developed HPLC protocol of Anderson and Taphouse, 1981 [34], as described in detail by Avnir et al. [10]. Liposome phospholipid concentration was determined using the modified Bartlett procedure [35].

#### Preparation of peptide conjugated NSSL (actively targeted NSSL)

EPC-based NSSL were prepared as described above, attached covalently to one of two lipidated peptides. One peptide is composed of 11 amino acids, 2162 Da, representing the binding site of Apo-lipoprotein E (ApoE) [36]; the other peptide is composed of 7 amino acids, 1626 Da, derived from Beta-amyloid [37]. The two peptides are attached covalently to dioleoyl (DO)-succinate to form the lipidated peptides [38]. These two putative peptides can be transported through the BBB. To make targeted BBB-penetrating NSSL each of the lipidated peptides was added to the lipidic ethanol solution (at 0.5% mole ratio) before preparation of the liposomes and hydrated together with other lipids used for targeted NSSL preparation. The efficacy of the transporter peptides to transport the NSSL and their payload through the BBB was tested using double labeling: Texas Red (red) for the liposomes, and calcein (orange) as a payload. After loading, excess calcein was removed by Sephadex column. Similar targeted HSPC-based NSSL remote loaded with MPS (as described above) were prepared for evaluating the therapeutic efficacy of the 2 different targeted NSSL.

#### Small angle X-ray scattering (SAXS)

For understanding membrane structure of the nano-liposomes, as well as drug interaction with the lipid membrane, and, most importantly, drug organization inside the liposomes, X-ray scattering measurements were made on NSSL loaded with drug, empty NSSL, free drug solution, and buffer solution. High-resolution SAXS measurements were taken at beamline 5.2 L at the ELETTRA synchrotron (Trieste, Italy). The X-ray photon energy was 8 keV; Si(111) was used as a monochromator; the beam size was set to 1000x500 μm^2^ (H and V fwhm, respectively); and a MAR300 image plate detector (Marresearch GmbH, Norderstedt, Germany) was used. In all of the measurements, silver behenate was used as a standard to determine the sample-to-detector distance. All the 2D images obtained had concentric rings resulting from the isotropic character of samples in solution. The images were radially integrated, using FIT2D [39], to obtain a 1D curve of the scattering intensity as a function of the magnitude of the momentum transfer vector, q. Data analysis was conducted using the X+ ‏software developed by Raviv and colleagues [40, 41].The chosen model for the electron density (ED) profile of the liposome membrane is a stack of infinite slabs with an asymmetric Gaussian electron density profile along the vertical direction. Owing to the liposome curvature, conformations of PEG and lipid headgroups might differ between the inner and outer bilayers of the membrane.

#### Differential scanning calorimetric (DSC) measurements

For understanding the thermotropic behavior of the NSSL composed of DMPC:DPPC:PEG-DSPE:Cholesterol in 24:30:5:41 mole ratio, or of the PCs alone, the desired lipid mixtures were hydrated in saline at 40 mM phospholipid concentration and characterized by differential scanning calorimetry (DSC) using MicroCal^TM^ VP-DSC GE Healthcare Life Sciences (Uppsala, Sweden). The scanning in the range of 10°C to 80°C at the rate of 1°C per min was done against saline as a reference. Data analysis was done using the Origin 7.0 software.

#### Magnetic resonance imaging (MRI)

In order to better understand the effect of NSSL-MPS treatment on the diseased brain pathology (edema, tissue composition, water diffusion, and BBB penetration) MRI was employed. Twelve mice were scanned in a 7T MRI system (Bruker, Germany) using a quadrature head coil. The mice were anesthetized with 0.5–1.5% Isoflurane and oxygen, and were maintained at °37C; their breathing was monitored with a breathing sensor.

**T2 protocol** was performed with the following T2 parameters: MSME sequence, TR = 3500 ms, 16 different equally spaced TE (ms) between 10 and 160; spatial resolution: 0.07x0.14x0.8mm. T2 map was generated for each mouse from the T2 weighted images using a nonlinear fit. Bias correction, spatial registration, and spatial normalization were performed using the SPM software. Ventricles analysis: Ventricles measurement analysis was performed using in-house MATLAB software. In each T2 map, the ventricles (ROI-region of interest) were marked in each slice and the total volume of the ventricles was calculated and normalized to whole brain size. Statistical analysis: *t*-test (between G0-naïve mice and G1-untreated mice) and one-way ANOVA (between the three groups), voxel based analysis (VBA) using in-house software written in MATLAB (Mathworks, USA). Significant clusters (P<0.01) are presented superimposed on a mouse atlas.

**Diffusion tensor imaging (DTI) protocol** was performed with the following DTI parameters: TR/TE = 5000/30 ms, 4 EPI segments, Δ/δ = 10/4.5 ms, 15 non-collinear gradient directions with a single b value shell at 1000 s/mm^2^ and one image with b value of 0 s/mm^2^ (referred to as b0). Geometrical parameters were: 20 slices of 0.8 mm thickness (brain volume) and in-plane resolution of 0.14x0.14mm^2^ (matrix size of 128x128 and FOV of 18 mm^2^). Apparent Diffusion Coefficient (ADC) maps were calculated in MATLAB using in-house software. For statistical comparisons between mice, each brain volume was normalized with a template mouse allowing voxel-based statistics. All image transformations were done using SPM (version 2, UCL, London, UK). Histogram analysis: histogram analysis is a whole brain analysis showing the distribution of the ADC parameter in the brain. ADC histograms were computed with 0.01 s/mm^2^ wide bins and normalized to the brain size. Statistical analysis: one-way ANOVA voxel based analysis (VBA) was performed using in-house software written in MATLAB between the three groups. Significant clusters (P<0.01) are presented superimposed on a mouse atlas. The ADC values from each brain were extracted in the clusters passing the statistical threshold and their averages are shown in the graphs.

**T1-GD protocol** was performed with the following parameters: FLASH sequence TR/TE = 298/3.33 ms and spatial resolution 0.1125x0.1125x0.8mm. The protocol included one T1 weighted image pre-GD injection and three T1 weighted images post-GD injections. The gadolinium was injected IP in a concentration of 0.3 mM.

#### Evaluation of the efficacy of BBB transporting peptides to cross the BBB and deliver drugs to the mice brains

Three healthy SJL/female mice groups were tested: (1) control–calcein loaded passively targeted NSSL treated group; (2) calcein loaded NSSL conjugated to short fraction of Beta-amyloid that is covalently conjugated to 1,2-dioleoyl-*sn*-glycero-3-succinate; (3) calcein loaded NSSL conjugated to short fraction of the binding site of Apo E that is covalently conjugated to 1,2-dioleoyl-*sn*-glycero-3-succinate. Each group included 4 mice. The mice were injected in the tail vein with an equal amount of calcein per kg body weight. Three hours later, mice were anesthetized, perfused with saline, and their brains were removed, fixed with paraformaldehyde in 30% sucrose for a week, and then frozen and serially sliced to 40-µm-thick frozen sections. Evaluation of the efficacy of the treatment was based on laser confocal microscopy of the brain slices. Observations were performed using specific lasers for activation of Texas Red and/or calcein.

#### Induction of acute EAE

Acute EAE was induced in 6-7-week-old SJL/J female mice as described previously [9, 11]. The animals were kept at the Hebrew University Animal Facility and given food and water ad libitum.

#### Induction of adoptive transfer EAE (AT-EAE)

8–10 days after EAE induction, lymph node cells were harvested and cultured at a density of 6 × 10^6^ cells/ml in DMEM [containing 10% FCS, 1% L-glutamine, 1% pyruvate, 1% antibiotics (penicillin—streptavidin), 5.10^-5^M β-mercaptoethanol, and 50 μg/ml PLP_139-151_. Cells were maintained in a humidified CO_2_ incubator at 37°C. Cells were harvested after 3 days and washed, and 1 × 10^7^–3 × 10^7^ cells were injected IV into naive 6–7-week-old SJL female mice. Clinical signs started to show up on days 7–8 post T-cell transfer, and maximal clinical signs appeared at about days 12–13.

#### EAE clinical scoring

The animals were monitored for clinical signs and were scored according to the following parameters: 0—Normal behavior, 1—Distal limp tail, 1.5—Complete limp tail, 2—Complete limp tail with righting reflex, 3—Ataxia, 4—Early paralysis, 5—Full paralysis, 6—Moribund/death. During the experiments, animals that exhibited clinical symptoms above ataxia were given saline as hydration fluid.

#### Statistical analysis

For each group, the mean daily clinical score is given in the figures and the tables, and from the combined data the following statistical parameters are given: mean maximal score, mean day of onset, and mean burden of disease (the mean of all the scores throughout all the days of the experiment). For mean burden of disease, Student’s *t-*test was used to determine statistical significance at P< 0.05.

## Results and Discussion

We designed two NSSL-based nano-drugs, each being remotely loaded with a drug aimed to treat diseases which involve inflammation, NSSL-GC [9, 12] and NSSL-TMN [11, 13]. Remote loading of liposomes by transmembrane ion gradients is one of the best approaches for achieving the high enough drug level per liposome required for therapeutic efficacy. Zucker et al. [42] and later Cern et al. [43] analyzed and characterized loading conditions taken from the data set generated during 15 years of research by the Barenholz laboratory on drug remote loading into liposomes [42] and from results of many other labs [43]. Two of the leading drugs in the data set used as successful examples for remote loading are the amphipathic weak acid steroid prodrug MPS, and the amphipathic weak base nitroxide TMN. Properties relevant to remote loading of these two APIs are described in **Table 1**.

**Table 1:** Physicochemical properties of NSSL-MPS and EPC-based NSSL-TMN.

	NSSL-MPS	EPC-based NSSL-TMN	DMPC:DPPC-based NSSL-TMN
**Drug pKa**	4.29^a^	8.9^b^	8.9^b^
**Drug LogD**	-0.47[at pH 7.6] ^a^	-3.6[at pH 6.7] ^b^, -3.11 [at pH 7] ^b^	-3.6[at pH 6.7] ^b^, -3.11 [at pH 7] ^b^
**Lipid composition**	HSPC:Chol:PEG-DSPE^c^	EPC:Chol:PEG-DSPE^c^	DMPC:DPPC:Chol:PEG-DSPE^d^
**Size (z-average; d.nm), PdI**	74.2±3.47, 0.042±0.014	74.4±6.3, 0.047±0.010	74.1±1.8, 0.030±0.012
**Ion gradient**	Calcium acetate, >1000	Ammonium sulfate, >1000	Ammonium sulfate, >1000
**Final mM drug**	12.7±1.4	7.0±0.65	7.5±0.14
**Drug-to-lipid mole ratio**	0.4±0.05	0.16±0.02	0.17±0.003
**% drug encapsulation**	>95%	>85%	>90%
**Storage stability (2–8°C)**	<6% drug release^e^	66% drug release^f^	6.23% drug release^f^

a- ACD/Labs.

b- Marvin. Values shown in each category are the average±STD for 6 different preparations. PdI: polydispersity index, an indication of variance in the sample. A low PdI (usually less than 0.2) indicates that the sample is monodispersed.

c- 54:41:5 mole ratio.

d- 24:30:41:5 mole ratio.

e- after 14 months [9].

f- after 4 months.

### Gross physicochemical properties of the nano-drugs fabricated

The features of the optimized NSSL-MPS, the EPC-based NSSL-TMN (used in previous studies) [9, 13], and the optimized NSSL-TMN based on a mixture of DMPC and DPPC are all presented in **Table 1**. The three formulations are successful examples of the remote loading approach, demonstrating high drug-to-lipid mole ratios and high loading efficiency, with the NSSL-MPS optimized formulation demonstrating a somewhat higher drug-to-lipid mole ratio (0.4±0.05 compared to 0.16±0.02 and 0.17±0.003 for EPC-based NSSL-TMN and DMPC:DPPC-based NSSL-TMN, respectively). The DMPC:DPPC-based NSSL show higher efficiency of encapsulation (>95% compared to >85% and 90% for EPC-based NSSL-TMN), also a better stability during 2–8°C storage (<6% drug release after 14 months compared to 66% drug release after 4 months for EPC-based NSSL-TMN, respectively), as well as demonstrating a slow, zero-order drug release kinetics in vivo [9]. These differences in loading efficiency and in stability can be explained by different API properties, especially their hydrophilicity, TMN being more hydrophilic than MPS (compare logD of -3.11 and -0.47 for TMN and MPS, respectively), and by drug interaction with the counterion used for the gradient formation [9]. The EPC-based NSSL used in previous studies were not viable for clinical application due to rapid TMN release at 2–8°C storage. NSSL membrane lipid composition determines to a large extent the stability and kinetics (reaction order and rate constant) of drug release [9, 13]. Wasserman et al. [13] reported that TMN release rate depends on the NSSL lipid composition and the type of ammonium salt counter anion, with ammonium sulfate being the slowest TMN-releasing formulation. Previously, we demonstrated that loading stability was improved when liposome-forming PCs having high T_m_, such as HSPC (T_m_ = 52°C) were used, but therapeutic efficacy of such NSSL was poor due to poor release at 37°C. The high resistance to release at 2–8°C is important for long shelf-life stability; however it may reduce the therapeutic efficacy due to a slower release rate at 37°C. The crucial role that NSSL membrane phosphatidylcholine species have on determining kinetic order and release rate of encapsulated drug is in agreement with previously reported results [9]. In order to try to improve shelf-life stability without compromising release rate under physiological conditions of 37°C, we have decided to study the effect of the intermediate-fluidity PCs by using PCs having shorter than C18:0 (stearoyl) chains. We selected DMPC (having two myristoyl, C14:0) and DPPC (having two palmitoyl, C16:0) and their mixtures in order to optimize the ratio of stability to release at physiological temperature and to achieve the optimal therapeutic efficacy. We designed a lipid membrane with a better profile of “free volume” to fit a fast enough release rate at 37°C concomitantly with a very slow drug release rate at 2–8°C storage temperature. For this we used a mixture of DMPC:DPPC:Chol:PEG-DSPE (24:30:41:5 mole ratio). The features of the optimized DMPC:DPPC-based NSSL-TMN studied here are also presented in **Table 1**, demonstrating an improved storage stability compared to the EPC-based NSSL-TMN, as well as somewhat higher loading efficiency.

### Effect of membrane lipid composition on TMN retention in NSSL

**Fig 1:**
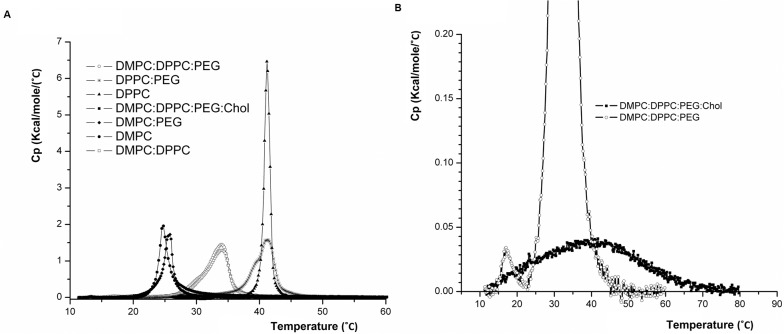
**(A)** DSC measurements. Samples of SUVs (DMPC:DPPC:Chol:PEG-DSPE, DMPC:DPPC:PEG-DSPE, DMPC:DPPC, DMPC:PEG-DSPE, DPPC:PEG-DSPE, DMPC, DPPC) in saline, and saline in the reference cell, were scanned in the range 10°-80°C, at the heating rate of 1°C/min. **(B)** Zooming in: Samples of SUVs DMPC:DPPC:Chol:PEG-DSPE, DMPC:DPPC:PEG-DSPE.

DSC measurements were done using SUV formulations of different lipid mixtures of DMPC, DPPC, PEG-DSPE, and cholesterol. As 33 mole% cholesterol is expected to abolish SO-to-LD phase transition of liposomal phospholipids [44], DSC measurements were done using an SUV formulation with the same lipid composition of DMPC:DPPC:PEG-DSPE (42.2: 52.8: 5 mole ratio) but without cholesterol. The thermotropic behavior assessed from the DSC scans presented in **Table 2**and **Fig 1A and 1B**demonstrates a phase transition range of 24.87 to 43.05°C; the endotherm has a half width of 4.0°C, a T_m_ of 34.2°C which is slightly below body temperature, and a relatively high phase transition enthalpy (*H)* of 6.961 kcal/mole. It is well established that for saturated PCs, ∆*H* increases with increasing PC acyl chain length and that the higher is ∆*H*, the stronger are the chain-chain associations [45, 46]. For comparison, DMPC-based SUV have a ∆*H* of 4.35 kcal/mole, while DPPC-based SUV have a ∆*H* of 8.2kcal/mole. DMPC:DPPC-based SUV demonstrate similar thermotropic behavior with a phase transition range of 24.8–43.5°C; the endotherm has a half width of 3.8°C, a T_m_ of 34.05°C, and a phase transition enthalpy (∆*H)* of 5.9 kcal/mole. This means that on storage this DMPC:DPPC-based nano-drug will resemble more the nano-drugs that are based on high-T_m_ PCs such as HSPC, and therefore will have a very slow drug release rate, which supports good storage conditions. The thermotropic behavior of DMPC:DPPC:PEG-DSPE:Chol assessed from the DSC scans is also presented in **Table 2**. To our surprise, at high lipid concentration there was a wide phase transition range (11.3–79.7°C) having a low phase transition enthalpy (*H* of 0.4 kcal/mole) and "width at half height" of 30.5°C. Namely, at 37°C these NSSL will be at the range of the SO-to-LD phase transition temperature, and therefore their LO phase will include enough free volume to enable sufficient release of TMN for therapeutic efficacy. However the 2–8°C storage temperature is below the temperature range of the nano-drug’s PCs phase transition.

**Table 2:** Thermotropic characterization of SUV aqueous dispersions assessed from the DSC scans.

	SUV aqueous dispersions						
Lipids	DMPC	DPPC	DMPC:DPPC	DMPC:PEG	DPPC:PEG	DMPC:DPPC:PEG	DMPC:DPPC:PEG:Chol
**Molar ratio (%)**	100	100	44.4/55.6	95/5	95/5	42.2/52.8/5	24/30/5/41
**Size (z-avarage, d.nm)**	60.22±0.9	62.46±3.6	59.05±0.3	62.73±0.4	61.77±0.5	71.31±0.7	74.26±0.1
	**Thermotropic characterization**						
**Tm (°C)**	23.48	41.168	34.05	25.189	41.39	34.22	43.27
**t1/2**	1.7	1.02	3.838	1.466	3.482	4.162	30.545
**ΔH (Cal.mol** ^**-1**^ **)**	5829	10144.5	5902.74	4551.44	6237.93	6961.91	438.03
**Range (°C)**	20.5–29	33.4–45.1	24.8–43.5	19.3–38.6	32.0–51.1	24.8–43.0	11.3–79.7
	**Fitting Model: MN2State**						
**Tm1 (°C)**	24.4±0.018	40.99±0.01	34.20±0.018	25.57±0.028	40.9±0.024	32.69±0.048	44.29±0.26
**ΔH1 (Cal.mol** ^**-1**^ **)**	4354±134	8246±139	1820±127	3588±97.0	5828±775	5013±110	456.1±7.74
**Tm2 (°C)**			32.55±0.073			34.24±0.016	
**ΔH2 (Cal.mol** ^**-1**^ **)**			4007±137			1886±102	

**Fig 2:**
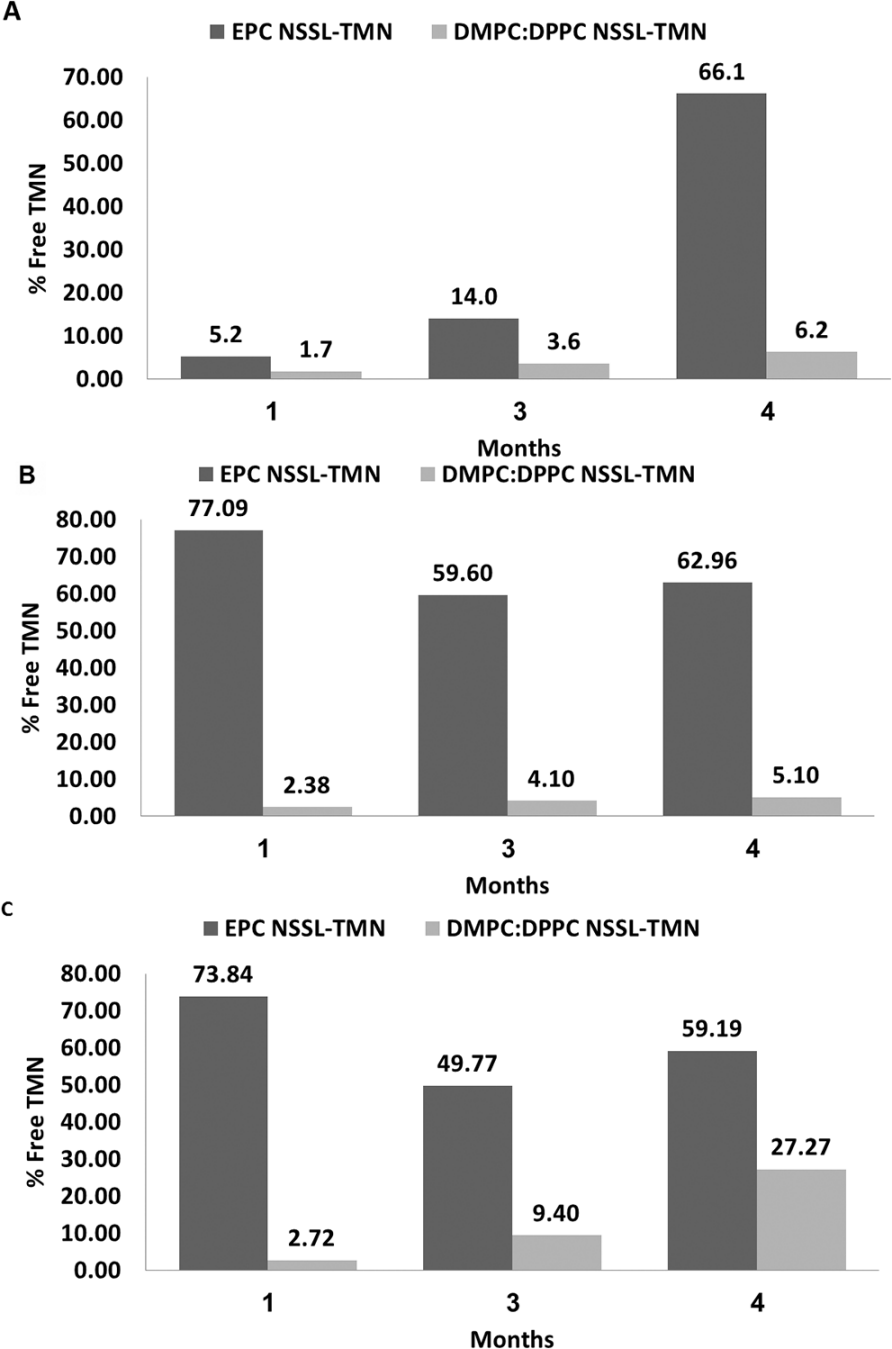
Effect of membrane lipid composition (EPC:Chol:PEG-DSPE or DMPC:DPPC:Chol:PEG-DSPE) on TMN retention in NSSL. % Free TMN was determined by ESR for both lipid compositions, EPC:Chol:PEG-DSPE (■) and DMPC:DPPC:Chol:PEG-DSPE (■) in vitro at 5°C **(A)**, 25°C **(B)**, and 37°C **(C)** during 4.5 months.

We determined TMN release in vitro at 5°C, 25°C, and 37°C during 4.5 months by ESR for both lipid compositions, EPC:Chol:PEG-DSPE (54:41:5 mole ratio) and DMPC:DPPC:Chol:PEG-DSPE (24:30:41:5 mole ratio) (**Fig 2A–2C**). Indeed, we found that this PC composition has clear benefits in vitro. EPC-based liposomes (having low T_m_ = -5°C and low SO-to-LD transition ∆*H)* support a fast release rate at 2–8°C storage (which is above EPC T_m_) with the majority of the drug (66%) released as free TMN. The DMPC:DPPC-based liposomes showed very similar release profiles at 25°C and 5°C as these are still below the T_m_ and the level of free volume is low, while at 37°C, release rate was higher than the release at 5 and 25°C. However, EPC-based liposomes released the majority of the drug (60–77%) at 25°C during the first month and reached a steady state. These results are in correlation to the T_m_ and ∆*H* of the liposomes made of DMPC:DPPC mixture 24:30 mole ratio (described above)_._ The release from EPC-based NSSL at 37°C was similar to the release at 25°C; more than 50% of the drug was released after the first month. Temperature is only one parameter affecting the release rate in vivo in the inflamed brain. There are probably additional local inflammation factors (e.g., cytokines, MMPs, etc.) that may support a more selective drug release in the diseased brain. The increased release rate of TMN at 37°C is an acceptable but not absolute indication of a better therapeutic effect.

### NSSL-TMN characterization by X-ray diffraction

The intensity measured in a solution X-ray scattering experiment is the Fourier transform of the electron density (ED) variation of nanoparticles in solution, squared and averaged on all possible orientations of the nanoparticles. Hence, solution X-ray scattering provides an indirect measurement of the structure and shape of nanoparticles in solution.

We have performed SAXS measurements on NSSL loaded with drug, empty NSSL, free drug solution, and buffer solution at two temperatures, 4 and 37°C. The electron density contrast of the NSSL membranes are modelled by a stack of five infinite layers having a Gaussian shape in the direction perpendicular to the membrane, representing, respectively, the inner and outer PEG layers, the inner and outer hydrophilic layers and the hydrophobic layer.

#### Membrane of NSSL composed of DMPC:DPPC

**Fig 3:**
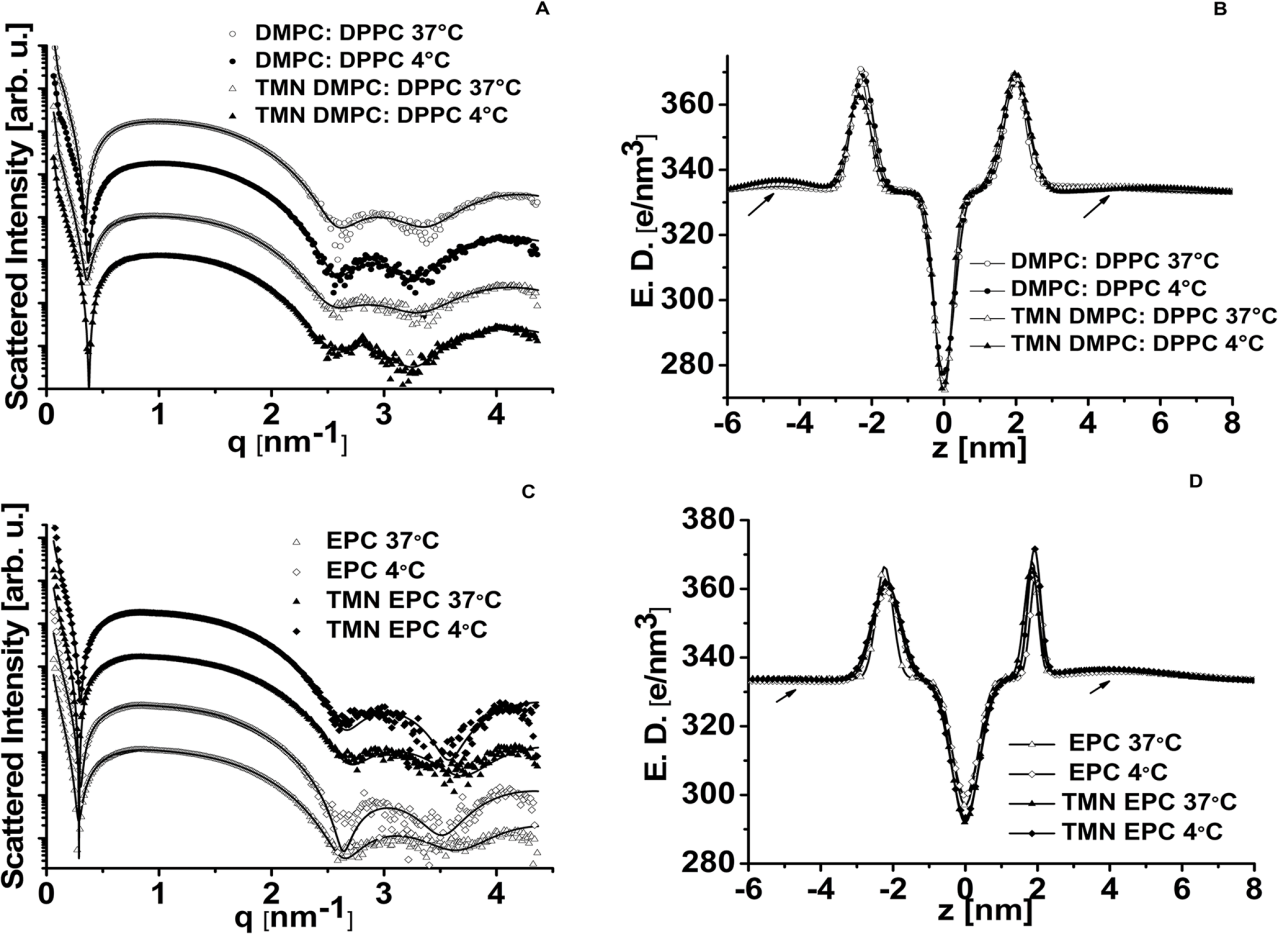
Small angle X-ray scattering (SAXS) measurements of NSSL-TMN. **(A)** Radially integrated background-subtracted scattering data (symbols) of DMPC:DPPC NSSL with and without drug, at 4 and 37°C, as indicated in the figure. Note that the curves are shifted in the intensity axis only for clarity of presentation. The solid curves are the corresponding form-factor models of a stack of infinite slabs with a Gaussian electron density profile along the vertical direction. **(B)** The electron density profiles of the DMPC:DPPC NSSL bilayers (with and without drug at 4 and 37°C) along the normal direction. The density profiles are obtained by fitting the scattering data to the models (see A) with the software X+, choosing a Gaussian electron density profile for the liposome membrane [40, 41]. The profile is almost symmetric and very slightly affected by the temperature or the presence of the drug. The arrows point to the profile of the inner and outer PEG layers. **(C)** The integrated scattering patterns as a function of the magnitude of the scattering vector, q, for EPC liposomes. Note that the curves are shifted in the intensity axis for clarity of presentation. The scattering curves of the EPC NSSL with and without drug, at 4 and 37°C are very similar. These curves are analyzed using the software X+, as in (A). The liposome bilayer is described by a Gaussian electron density profile. **(D)** The electron density profile in the direction normal to the membrane, calculated using the software X+, is presented for EPC NSSL, with and without drug at 4 and 37°C. The density profile of the membrane is almost unaffected by the temperature or the presence of the drug. Notice that this profile is asymmetric, suggesting that the inner and the outer PEG layers (pointed by an arrow) of the liposome are different.

**Fig 3A and 3B**show the scattering curves and the electron density (ED) profiles of NSSL membrane, loaded and unloaded with TMN, at 37 and 4°C. Before fitting the scattering curves, we subtracted the background scattering (due to the solvent, the capillary, and the presence of other dissolved molecules). As in our earlier studies, for background we used a power law [40] to account for the contribution of concentration fluctuations in the sample [47].

The membrane, as depicted by the ED profiles obtained from fitting the SAXS curves, is almost symmetric, whereas the outer and inner PEG layers are different. No noticeable difference is visible in the profiles of the membrane at 4 and 37°C. The membrane width, that is to say the head-to-head (phosphate-to-phosphate) distance obtained from the fit is 4.25 nm for both temperatures (**Table 3**). The shapes and sizes of the membrane are almost the same with and without TMN, a good indication that little or no drug has partitioned into the membrane, as expected according to the molecular properties of the drug. The lack of effect of TMN on the liposomal membrane was also confirmed by lack of effect of TMN remote loading on the thermotropic characteristics of SUV composed of DMPC:DPPC:PEG.

**Table 3:** Membrane width (head-to-head distance) of the NSSL at 4°C and 37°C, with and without TMN using small angle X-ray scattering measurements.

DMPC:DPPC-based NSSL				EPC-based NSSL			
37°C		**4°C**		**37°C**		**4°C**	
Empty	**TMN**	**Empty**	**TMN**	**Empty**	**TMN**	**Empty**	**TMN**
4.25	4.25	4.26	4.31	4.10	4.03	4.14	4.09

#### Membrane of NSSL composed of EPC

The EPC-based NSSL solution scattering curves at 4 and 37°C are shown in **Fig 3C**. They are fitted with the same model of asymmetric bilayer decorated on both sides with PEG. The electron density profile resulting from the fitting is presented in **Fig 3D**. The temperature slightly affects the width of the EPC-NSSL membrane (**Table 3**). The profile of the EPC liposome’s membrane loaded with TMN is less asymmetric than the profile of the liposome’s membrane without TMN, and the membrane width is smaller when the drug is entrapped in the vesicle. Hence, the introduction of the drug in the EPC-based NSSL changes the disposition of the components of the NSSL membranes, and the differences shown in the ED profile can be associated with a "pressure" exerted by the drug on the inner leaflet of the liposome membrane moving the lipids to the outer leaflet of the NSSL. The distribution of nitroxides into the lipid bilayer is dependent on their membrane-to-medium partition coefficient. For example, the more hydrophilic nitroxide Tempol (logD 0.22) has about a 10-fold lower concentration in the bilayer of EPC-based SUV compared to Tempo (LogD ~2). As TMN is even more hydrophilic (logD -3.11), its partition coefficient is expected to be lower, and therefore not to change the features of the liposomal membrane.

A comparison between the profiles of the membranes of EPC-NSSL DMPC:DPPC-based NSSL shows that the EPC-NSSL membrane is more asymmetric and its width is smaller. This can be explained by the double bonds in the EPC tails leading to a more fluid membrane, reflected in a higher drug release rate and lower storage stability [16].

### Comparison of the therapeutic efficacy of EPC:Chol:PEG-DSPE NSSL-TMN and DMPC:DPPC:Chol:PEG-DSPE NSSL-TMN

We have previously shown that EPC-based NSSL-TMN is efficient in inhibiting EAE in mice, as well as adjuvant induced arthritis in rats [11, 13, 48]. When comparing treatment in the acute EAE mouse model with the optimized NSSL-MPS nano-drug (50mg/kg) and the previously described EPC-based NSSL-TMN (8.5mg/kg) in terms of therapeutic efficacy, both treatments show significant therapeutic efficacy compared to the control (saline) group, with the NSSL-MPS treatment resulting in somewhat lower disease burden (and therefore having better therapeutic efficacy) compared to the EPC-based NSSL-TMN treatment (**S1 Fig and S1 Table**). Kizelsztein et al. showed that EPC-based NSSL-TMN significantly ameliorates the clinical symptoms of EAE, both in its acute and chronic forms. The penetration of EPC-based NSSL-TMN into the brain was 3 to 4-fold higher in EAE mice than in normal mice, and free TMN could not reach therapeutic efficacy in the diseased brain due to its rapid clearance [11].

**Fig 4:**
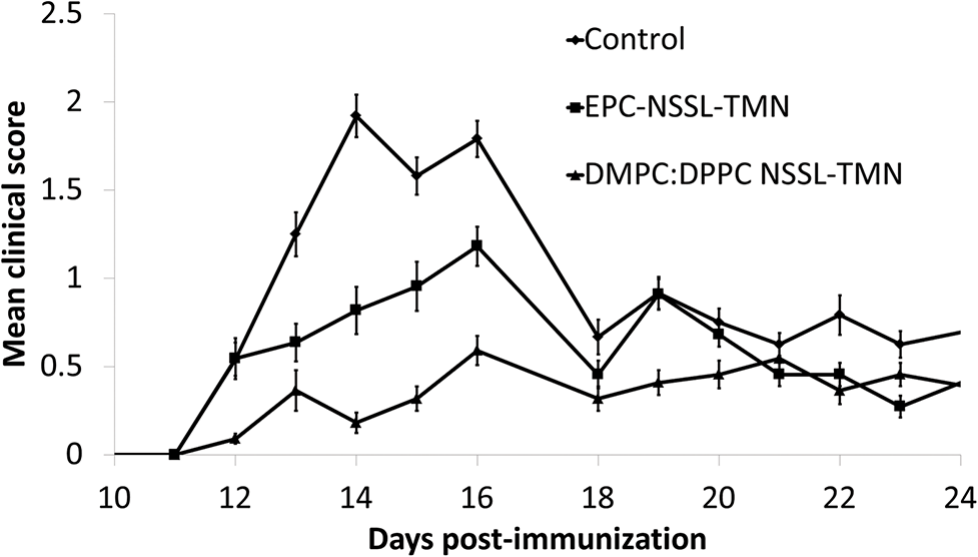
Comparison of the therapeutic efficacy of EPC:Chol:PEG-DSPE NSSL-TMN and DMPC:DPPC:Chol:PEG-DSPE NSSL-TMN in acute EAE mice model. SJL/J mice (n = 10) were treated by IV injections every other day starting on day 8 with: EPC:Chol:PEG-DSPE NSSL-TMN 8.5 mg/kg BW (■), DMPC:DPPC:Chol:PEG-DSPE NSSL-TMN 8.5mg/kg BW (▲), and dextrose 5% (control) (●).

The DMPC:DPPC-based NSSL-TMN nano-drug demonstrated an improved storage stability at 2–8°C compared to the EPC-based NSSL-TMN nano-drug. However, the high resistance to release may reduce the therapeutic efficacy due to a slower release rate at 37°C (**Fig 2C**). When comparing the therapeutic efficacy of DMPC:DPPC-based NSSL-TMN and EPC-based NSSL-TMN in acute EAE mice, we demonstrated that not only was the less "fluid", optimized DMPC:DPPC formulation therapeutically active, it actually showed a better and prolonged therapeutic effect compared to the EPC-based NSSl-TMN. DMPC:DPPC-based NSSL-TMN demonstrated superior therapeutic efficacy compared to the control group and EPC-based NSSL-TMN- treated group (mean burden of disease of 0.26±0.05 compared to 0.77±0.08 and 0.5±0.07, respectively, **Fig 4, Table 4**). The lower leakage rate of TMN from DMPC:DPPC-based NSSL than from EPC-based NSSL (**Fig 2**) might explain the in vivo benefits of DMPC:DPPC-based NSSL-TMN. This formulation showed reduced amounts of TMN release in vitro at 37°C, and therefore probably lower “leakage” into the blood circulation, leading to a high TMN level at the disease site and better therapeutic efficacy compared to EPC-based NSSL. EPC-based liposomes (having low T_m_ of -5°C and low SO-to-LD transition ∆*H)* are characterized by a lipid bilayer having high compressibility [49], namely, the LO phase membrane has a much larger number of free volume defects [49–52]. This free volume is what determines the kinetic order and rate of drug release. In short, in NSSL based on high-T_m_ PCs, the small number of free volume defects becomes saturated already at low drug concentration, and consequently the system operates under conditions of Vmax, which results in a slow, zero-order release. In terms of medical application, if a liposome bilayer in the LO phase is composed of a phospholipid with a T_m_ much below 37°C, like EPC, when these liposomes are used in vivo at 37°C the membrane will have high compressibility due to the high level of membrane free volume. This will allow encapsulated agents (in our case TMN) to be released faster than from liposomes with membranes based on liposome-forming lipid with T_m_ above 37C, where even at the LO phase the free volume is small.

**Table 4:** Comparison of the therapeutic efficacy of EPC-based NSSL-TMN and DMPC:DPPC-based NSSL-TMN in acute EAE mice model.

Group	Incidence (#dead)	Mean maximal score	Mean onset of disease (day)	Mean duration (days)	Mean burden of disease
**Control**	10/12 (0)	2.45±0.28	13±0.25	9.2±1.1	0.77±0.08
**EPC NSSL-TMN**	8/11 (0)	2.2±0.37	16±1.4	7.2±1	0.5±0.07
**DMPC:DPPC NSSL-TMN**	7/11 (0)	2.1±0.32	17.6±1.7	5.5±1	0.26±0.05 ^a^ ^,^ ^b^

^a^ Significant difference from the control group P<0.0001

^b^ Significant difference from the EPC NSSL-TMN treated group P<0.001.

Altogether, the DMPC:DPPC-based NSSL-TMN was found to be more suitable for clinical application, taking into consideration the improved stability and improved therapeutic efficacy in the EAE model. We suggest that a similar kind of optimization procedure should be done for every developed liposomal drug. Such a procedure should be fitted to the drug’s unique physicochemical properties, the first and relatively "simple" parameter of such optimization being the exact composition of the liposome-forming PCs.

### Animal studies of NSSL-MPS nano-drug

#### Comparison of the therapeutic efficacy of 50 and 10mg/kg NSSL-MPS in the acute EAE mice model

**Fig 5:**
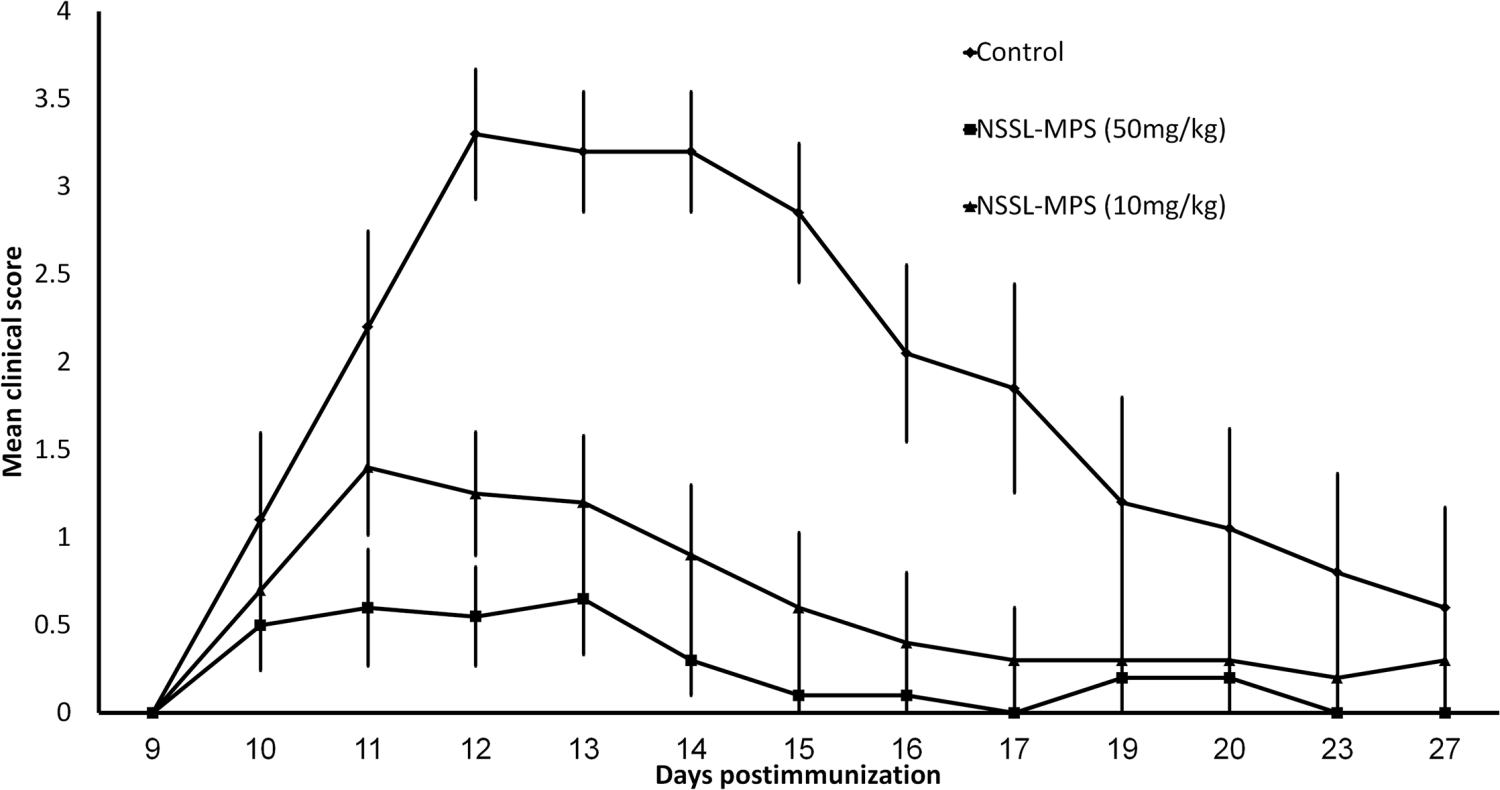
Comparison of the therapeutic efficacy of 50 and 10mg/kg NSSL-MPS in the acute EAE mice model. SJL mice were treated by IV injections on days 10, 12, 14 post-immunization with saline (control) (◆), 10mg/kg NSSL-MPS (▲) or 50mg/kg NSSL-MPS (■).

We have shown that treatment with 50mg/kg NSSL-MPS administered IV in the acute EAE mice model had superior therapeutic efficacy than free MPS and currently used drugs (Betaferon and Copaxone)[9]. In Fig 5 we compared the therapeutic efficacy of 50 and 10mg/kg NSSL-MPS in the acute EAE mice model. Treatment with NSSL-MPS showed significant therapeutic efficacy even at 5-fold lower dosage (10mg/kg compared to 50mg/kg) and the mean burden of disease was significantly lower for both treatment groups compared to control group (Fig 5, Table 5). Our results are in agreement with previously published dose titration experiments underscoring a dose-dependent efficacy of liposomal GC (using prednisolone phosphate as the active drug instead of MPS prodrug) with a sustained efficacy especially of the higher dosage (10mg/kg, but not at 4mg/kg) in MOG-EAE DA rats [19]. While the optimal dosage for GC pulse in treatment of MS is still under debate, many studies point to a dose-dependent efficacy of free GC. At a lower concentration, GC effects are mainly mediated by the classical nuclear GC receptor, but at a higher concentration, additional, non-genomic mechanisms may be operative, such as through membrane receptors (for example, G-protein-coupled receptors [53]) and activation of a second messenger system [54, 55]. These pathways are thought to be one possible explanation for the observed superiority of high and ultra-high doses in the treatment of some autoimmune disorders [56]. Although tissue targeting by NSSL formulations already results in higher local tissue concentrations, dose-dependency is apparently of similar importance for the action of liposomal GC as for free GC.

**Table 5:** Comparison of the therapeutic efficacy of 50 and 10mg/kg NSSL-MPS in the acute EAE mice model.

Group	Incidence (#dead)	Mean maximal score	Mean onset of disease	Mean duration (days)	Mean burden of disease
**Control**	11/11 (2)	4.2±0.35	10.8±0.26	9.5±1.3	2.26±0.18
**NSSL-MPS 50mg/kg**	5/10 (0)	1.1±0.24 ^a^	12.4±1.2	1.9±0.8 ^c^	0.26±0.06 ^a^
**NSSL-MPS 10mg/kg**	10/10 (0)	1.9±0.4 ^a^	10.9±0.27	4.7±1.5 ^a^	0.65±0.1 ^a^ ^,^ ^b^

a- Significant difference from the control group P<0.0001

b- Significant difference from NSSL-50mg/kg group P<0.005

c- Significant difference from the control group P<0.05.

#### Comparison of the therapeutic efficacy of passively targeted NSSL-MPS and actively targeted peptide-conjugated NSSL-MPS in the acute EAE mice model

**Fig 6:**
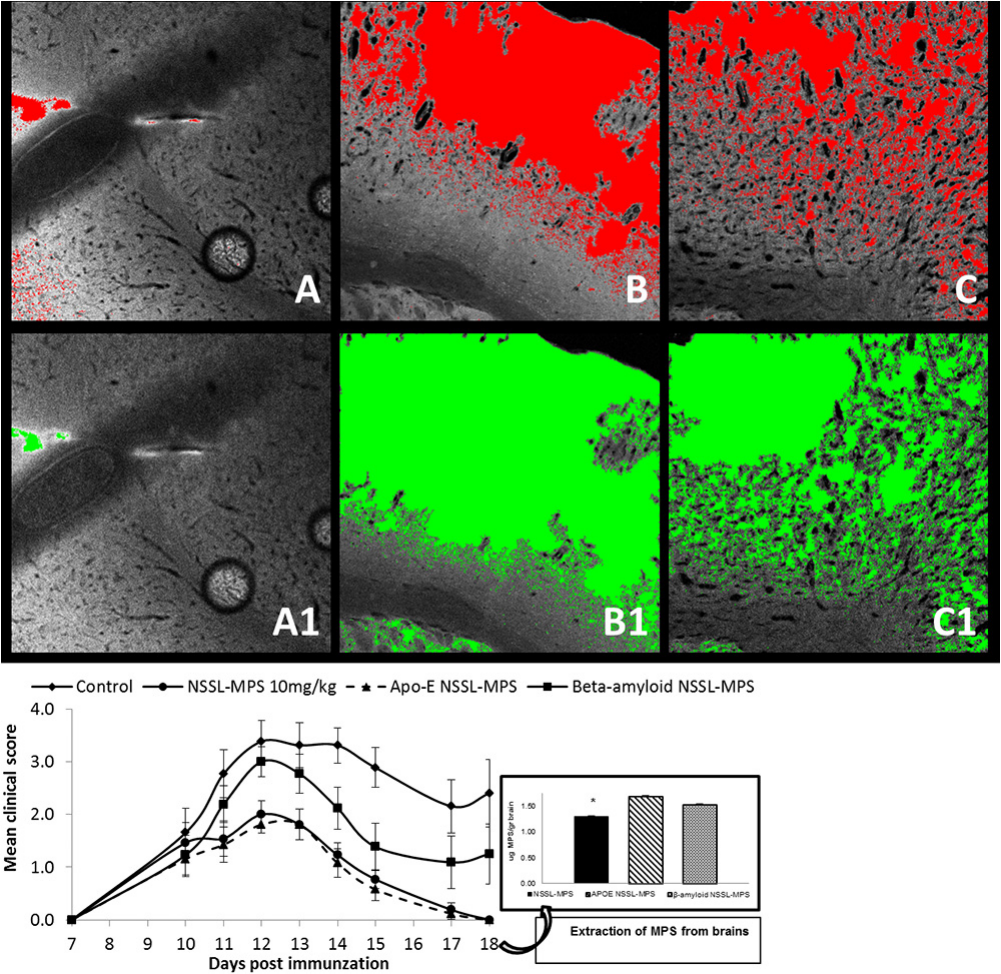
Comparison of passively targeted NSSL and actively targeted peptide-conjugated NSSL. **(A)** Representative fluorescent microscopy images comparing brain accumulation of NSSL and their payload as is (A, A1), β-amyloid NSSL(B,B1), and ApoE NSSL (C,C1) in healthy mice brain showing an increase in the amount of actively targeted NSSL and their payload accumulating, compared to passively targeted NSSL. **(B)** Comparison of the therapeutic efficacy of passively targeted NSSL-MPS and actively targeted peptide-conjugated NSSL-MPS in the acute EAE mice model. SJL mice were treated by IV injections on days 10, 12, 14 post-immunization with saline (control) (◆), NSSL-MPS (●), Apo-E NSSL-MPS (▲) or β-amyloid NSSL-MPS (**■**). * p-value < 0.0001.

To answer the question whether treatment with actively targeted NSSL-MPS (as opposed to the passively targeted NSSL-MPS) will further improve the therapeutic efficacy of these drugs we fabricated actively targeted NSSL-MPS, presenting peptides that enable them to cross the blood-brain barrier and release their payload in the brain. As the targeting ligands we used short peptide fragments of ApoE or of β-amyloid that were pre-conjugated to dioleoyl succinate (DO-succinate). Preliminary results using NSSL liposomes labeled with Texas Red and loaded with calcein showed increases in the amount of actively targeted liposomes and their payload accumulated in healthy mice brains after injecting these mice with actively targeted liposomes, compared to passively targeted liposomes. Fig 6A shows representative fluorescent microscopy images comparing brain accumulation of NSSL and their payload: NSSL as is (A, A1); β-amyloid NSSL (B,B1); and ApoE NSSL (C,C1) in healthy mice brain showing an increase in the amount of actively targeted NSSL and their payload accumulated compared to passively targeted NSSL. We further compared the therapeutic efficacy of these targeted NSSL loaded with MPS in the acute EAE mice model (Fig 6B, Table 6). SJL mice were injected on days 10, 12, 14 post immunization with saline (control) or 10mg/kg NSSL-MPS (with or without the targeting peptides attached to their surface). Gaillard et al. [57] tried to enhance brain penetration of NSSL-MPS by using a targeted NSSL-MPS formulation (based on our previously published remote loaded NSSL-MPS formulation conjugated to the brain-targeting ligand glutathione), demonstrating a slight advantage (at best) in the MBP-EAE rats over the passively targeted formulation, apparent only 24 h after treatment. In the present study, although at the end of the experiment, a week after the third and last injection, MPS levels were significantly higher in the targeted liposomes-treated groups compared to the NSSL-MPS-treated group (Fig 6B). Both targeted formulations (ApoE- and β-amyloid-conjugated NSSL) did not show any therapeutic advantages over the passively targeted NSSL formulation, although all three formulations significantly ameliorated disease severity compared to control group. Furthermore, β-amyloid NSSL-MPS treatment was significantly less efficacious than passively targeted NSSL-MPS treatment. Treatment with ApoE NSSL-MPS showed similar therapeutic efficacy to treatment with NSSL-MPS (mean burden of disease: 0.9±0.1 and 1±0.1, respectively) compared to control (mean burden of disease 2.4±0.17). Treatment with β-amyloid NSSL-MPS lowered slightly the mean maximal clinical scores (3.2±0.31) and the mean burden of disease (1.7±0.14) compared to the control group (3.9±0.35, 2.4±0.17). Therefore, the improved penetration into the brain did not correlate with improved efficacy. The actively targeted NSSL probably distribute less uniformly throughout the diseased brains while remaining attached to cells close to the blood vessels, similarly to what was demonstrated before for tumor targeted NSSL [58]. In addition, BBB disruption in EAE mice, which enables penetration of passively targeted NSSL, might explain the lack of therapeutic advantage of actively targeted NSSL which may be able to extravasate the intact BBB over the passively targeted NSSL.

**Table 6:** Comparison of the therapeutic efficacy of passively targeted NSSL-MPS and actively targeted peptide conjugated NSSL-MPS in the acute EAE mice model.

Group	Incidence (#dead)	Mean maximal score	Mean onset of disease	Mean duration (days)	Mean burden of disease
**Control**	13/13 (2)	3.9±0.35	10.8±0.32	7.5±0.38	2.4±0.17
**NSSL-MPS**	13/14 (0)	2.5±0.30 ^e^	10.5±0.23	4.8±0.57 ^b^	1.0±0.10 ^a^ ^,^ ^c^
**ApoE NSSL-MPS**	14/14 (0)	2.5±0.27 ^e^	10.6±0.20	4.5±0.52 ^f^	0.9±0.10 ^a^ ^,^ ^d^
**β-amyloid NSSL-MPS**	14/14 (1)	3.2±0.31	10.5±0.17	6.1±0.59	1.7±0.14 ^b^

^a^ Significant difference from the control group P<0.00001

^b^ Significant difference from the control group P<0.001

^c^ Significant difference from the β-amyloid NSSL-MPS group P<0.0005

^d^ Significant difference from the β-amyloid NSSL-MPS group P<0.0001

^e^ Significant difference from the control group P<0.005

^f^ Significant difference from the control group P<0.0001.

#### Comparison of the therapeutic efficacy of NSSL-MPS and free MPS in the adoptive transfer EAE mice model

**Fig 7:**
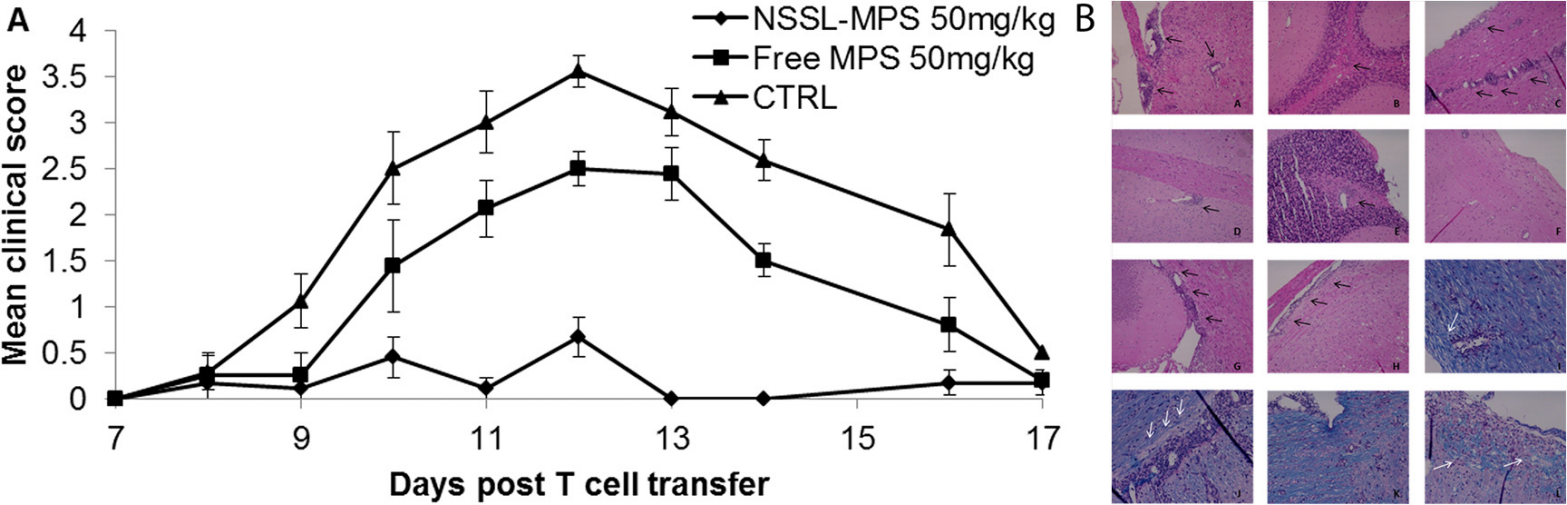
Comparison of the therapeutic efficacy of NSSL-MPS and free MPS in the adoptive transfer EAE mice model. **(A)** SJL mice were treated by IV injections on days 8, 10, 12 post-T cell transfer with saline (control) (▲), free MPS (50mg/kg) (■) or NSSL-MPS (50mg/kg) (◆). **(B)** Treatment with NSSL-MPS reduced inflammation and demyelination in brains and spinal cords of mice with AT-EAE compared with free MPS treated mice and control mice. Brains and spinal cords were obtained on day 13 post T- cell transfer. Black arrows indicate infiltrating inflammatory cells; white arrow indicates demyelination. Representative H&E-stained brains (A,B,D,E,G) and spinal cord (C,F,H) sections from control (A-C), as well as NSSL-MPS (D-F) and free MPS treated EAE mice (G-H) show extensive inflammation involving perivascular infiltrates of mononuclear leukocytes (arrows) within the cerebral parenchyma and spinal cords of CTRL mice and free MPS treated mice (arrows). In the NSSL-MPS treated group, much fewer infiltrating cells were observed. LFB staining demonstrates extensive demyelination in the control spinal cord (J) and a decrease in myelin density in the free MPS treated group (I,L) around blood vessels compared with NSSL-MPS treated mice (K), demonstrating densely organized myelin sheaths. Original magnification of x 40.

As an alternative to direct induction with PLP, EAE can also be induced in SJL mice by adoptive transfer of in vitro neuroantigen-activated lymphocytes from mice immunized with these encephalitogenic neuroantigens. Induction of EAE by this method usually results in more severe disease, with higher incidence and a more accelerated and synchronous disease course. This model is a very sensitive therapeutic model of EAE, and was used extensively in MRI studies [47, 59]. We tested the therapeutic efficacy of our NSSL-MPS formulation compared to the free MPS drug under stringent conditions, starting treatment at the time of first clinical signs of EAE (Fig 7A, Table 7). We compared the therapeutic effect of NSSL-MPS and the free MPS drug. Mice were injected on days 8, 9, and 10 after T-cell transfer with the onset of clinical signs. For both treatment groups, the liposomal and the free MPS, the mean maximal score disease duration and the mean burden of disease were significantly improved compared to control group. Although treatment with free MPS showed clear efficacy, treatment with the liposomal MPS was much more effective. At day 13 after T-cell transfer, we examined the brains and spinal cords of mice from each treatment group (CTRL, free MPS, and NSSL-MPS) using luxol fast blue (LFB) and hematoxylin and eosin (H&E) staining as indications of demyelination and leukocyte infiltration, respectively, to determine whether the differences in therapeutic efficacy correspond to differences in neuropathology (Fig 7B). H&E staining clearly showed inflammation and accumulation of infiltrated cells in the brain and spinal cord. In both the control group (sham-treated) and the free MPS-treated group, cellular infiltrates were dense in the parenchyma and perivascular spaces, brainstem, cortex, and cerebellum.

**Table 7:** Comparison of the therapeutic efficacy of NSSL-MPS and free MPS in the adoptive transfer EAE mice model.

Group	Incidence (#dead)	Mean maximal score	Mean onset of disease	Mean duration (days)	Mean burden of disease
**Control**	9/9 (0)	3.7±0.16	9.1±0.26	7.0±0.57	1.9±0.15
**NSSL-MPS**	6/9 (0)	0.83±0.22 ^a^	11.3±0.91	1.7±0.57 ^c^	0.2±0.05 ^a^
**Free MPS**	8/8 (0)	2.83±0.31 ^b^	10.1±0.35	2.3±0.80 ^d^	1.2±0.13 ^d^ ^,^ ^a^

^a^ Significant difference from the control group P<0.000001

^b^ Significant difference from the NSSL-MPS group P<0.0005

^c^ Significant difference from the control group P<0.00005

^d^ Significant difference from the NSSL-MPS group P<0.005.

In the NSSL-MPS-treated mice, although infiltrating cells were present, many fewer were observed. This may indicate that infiltration of new cells during disease progression may be an important effect of this treatment. McCombe et al. studied the effects of high dose corticosteroid treatment on the numbers of lymphocytes obtained from the spinal cords of Lewis rats with EAE. In these rats there was a reduction in the numbers of T-lymphocytes in the spinal cord [60]. LFB staining showed areas of demyelination and a decrease in myelin density in brains and spinal cords of mice from the control and free MPS-treated groups, while in the brains and spinal cords of NSSL-MPS treated mice, densely organized myelin sheaths were observed. The reduction in T-cell infiltration of the nervous system could lead to clinical benefits by reducing ongoing demyelination (as was demonstrated by LFB staining), thus allowing the remyelination that normally occurs during recovery from EAE to restore neurological functions.

#### Characterization of acute EAE mice using magnetic resonance imaging (MRI)

**Fig 8:**
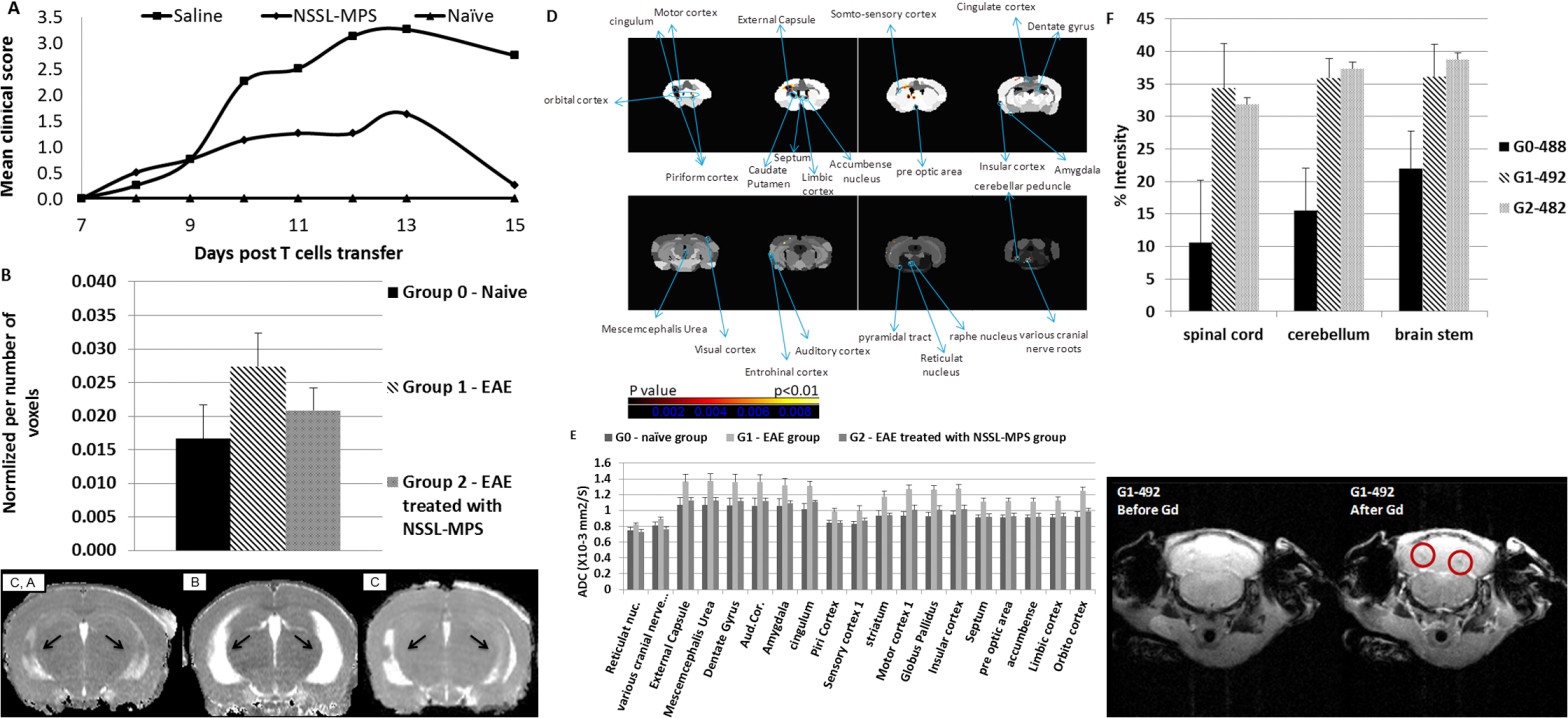
Brain tissue characteristics of acute EAE mice using magnetic resonance imaging. **A:** The therapeutic efficacy of NSSL-MPS (10mg/kg) in the adoptive transfer EAE mice model. SJL mice were treated by IV injections on days 8, 10, 12 post-T cell transfer with saline (control) (■), or NSSL-MPS (10mg/kg) (◆) and were scanned in a 7T MRI system on day 16 after T-cell transfer. **B**: Ventricles volume measurement normalized to total brain volume. The ventricles volume in the EAE group (G1) was bigger than the volume in the naïve and NSSL-MPS (10mg/kg) treated groups (G0 and G2). **C**: T2 images of representative mice brains. (A) G0 –naïve group, (B) G1 –EAE group, (C) G2 –EAE treated with NSSL-MPS (10mg/kg) group. **D**: Voxel-based one-way ANOVA between naïve, treated with NSSL-MPS, and untreated group. The significant clusters are presented on a brain atlas. **E**: Graphs of the ADC value in each significant cluster in each group. In all regions, ADC value is higher in the EAE group (G1) compared to the treated (G2) and naive group (G0). **F**: Representative mouse brain from each group (G0-488; G1-492; G2-482) with the % intensity of change after Gd injection in the spinal cord, cerebellum, and the brain stem. **G**: Cerebellum slices of G1-492 mouse before and after Gd injection. Lesions appearance after Gd injection, indicate by reduced of signal marked in red in the image.

The therapeutic effect was accompanied with changes in the brain tissue characteristics compared to control group observed by MRI (Fig 8A–8G). A short course of high-dose intravenous methylprednisolone (IVMP) is widely used to treat clinical relapses in MS [61–64]. The mechanism of action of IVMP, as of other forms of steroids, is uncertain. Suggestions have included the reduction of edema [65], immunosuppression [15], direct inhibition of demyelination [66], direct effect on axonal conduction, and reversal of blood-brain barrier (BBB) abnormalities [67]. MRI is increasingly used for diagnosing multiple sclerosis and assessing disease prognosis [68]. It has provided evidence regarding the therapeutic efficacy of steroids in MS relapses. In this study, mice were treated with 10mg/kg NSSL-MPS or saline (control) on days 8, 10, and 12 after T-cell transfer and were scanned in a 7T MRI system on day 16 after T-cell transfer. Fig 8B and 8C represents the average ventricles volume normalized to total brain volume in each group. T2 relaxation maps are suited to image edema and can also be used for measurement of brain ventricles volume. The ventricles measurements showed significantly larger ventricles in the EAE group compared to the naïve and treated mice. This pathology was previously indicated in the EAE model [69]. In diffusion tensor imaging (DTI) we can estimate the trace of the diffusion tensor or average diffusivity (ADC), a putative measure of edema or reduced tissue integrity. ADC maps showed a higher ADC parameter in the EAE group compared to the naïve and the treated groups (Fig 8D). This indicates that the diffusion of water molecules in the EAE brain tissue is higher and might result in changes in cellular/axonal damage and reduced tissue integrity. One-way ANOVA analysis shows several regions with significantly higher ADC in the EAE group compared to the naïve and treated groups (Fig 8E). This shows that in specific regions the differences in gray and white matter integrity were observed in the EAE model and following treatment. Extraction of ADC parameters in the significant clusters demonstrated higher ADC value in the EAE group compared to the naïve and treated groups. Such changes may occur due to increased tissue repair, including increased myelin density (as we have seen in LFB staining, Fig 7B), as well as increased activation and gliosis (microglia/astrocyte) in the EAE treated group (lower ADC parameters). T1-weighted images were performed before and after injection of gadolinium (Gd). Areas of Gd enhancement demonstrated on T1-weighted MRI are believed to reflect underlying blood-brain barrier disruption at active perivascular infiltrate and inflammation. Fig 8F shows a representative mouse brain from each group (G0-488 naïve; G1-492 EAE untreated; G2-482 EAE treated with NSSL-MPS) with the percentage intensity of change after Gd injection in the spinal cord, cerebellum, and the brain stem. There were higher enhancements of the signal (30–40% intensity) in both represented brains from treated and untreated groups (G1-492 and G2-482); in the mouse from the naïve group (G0-488) there was a slighter enhancement (10–20% intensity). This Gd enhancement may indicate a BBB penetration/breakdown/ [70]. There are conflicting reports regarding BBB disruption as a consistent component in the development of EAE and in the sequence of events leading to the development of signs of disease. Some studies have indicated that vascular permeability is a distinct event that precedes cellular infiltration [6, 71, 72], while other studies have found BBB permeability to be present only during inflammation [73–75]. Though these observations may be dependent on the specific model of EAE, the role of BBB permeability in the initial development and during the progression of the disease is unclear. Schmidt et al. [20] studied the effect of pegylated liposomes encapsulating prednisolone (PL) in the adoptive transfer EAE rat model. BBB integrity was determined by immunohistochemical staining for albumin. BBB disruption was greatly reduced by 10 mg/kg PL, which was superior to a 5-fold higher dose of free methylprednisolone (MP) on day 5 after cell transfer. Previously, serial iodine-enhanced CT scanning had shown that IVMP (high dose intravenous methylprednisolone) may rapidly reverse BBB abnormalities in MS lesions [76]. Treatment with IVMP showed a rapid reduction of BBB abnormalities within hours of administering methylprednisolone [76, 77], however the putative BBB abnormality may later reappear and many lesions re-enhanced within a few days of stopping IVMP. Miller et al. [77] reported that on day 7 after IVMP treatment, many lesions displayed increased enhancement in comparison with day 3, while at the same time all patients were clinically stable or improving. Therefore, given the therapeutic efficacy of 10mg/kg NSSL-MPS under the conditions employed (starting treatment at the onset of clinical signs and not shortly after T-cell transfer), it is reasonable to assume that the BBB was restored as a consequence of the treatment. Indeed, H&E staining demonstrated fewer infiltrating cells in the brains and spinal cords of EAE mice that were treated with NSSL-MPS compared to the untreated group, as well as to the free MPS-treated group (Fig 7B). However, structure abnormalities were visualized, an effect that can be explained by the re-appearance of BBB leakage due to the 4-day lag time until the scanning process. Even so, it is important to remember that there are pathophysiological mechanisms in MS relapses that are independent of the BBB, and that may or may not be modified by steroids, for example, immunological changes. Fig 8G demonstrates a cerebellum slice before Gd injection (left) and after (right). The marked regions indicate reduced signal in the suspected lesions. After Gd injection, one animal from the EAE untreated group (G1-492) seemed to show appearance of lesions. The appearance of lesions in the EAE untreated group may indicate the pathology of the disease [78]. Clinically, lesions that show such contrast enhancement on MRI are often identified as active lesions, and in many cases these lesions are found to correlate with clinical symptoms [79, 80].

## Conclusions

Brain pathology, as in the case of neuro-autoimmune diseases, is worsened, not only by the infiltration of activated lymphoid cells and by a high level of inflammatory cytokines, but also by the release of large amounts of free radicals [21]. Here we address these harmful elements on two fronts with two different NSSL drugs: NSSL-MPS and NSSL-TMN.

1. To neutralize free radicals there is a need for a potent antioxidant with long circulation time that can penetrate the diseased brain and be active there. We have previously shown that NSSL-TMN is efficient in inhibiting EAE in mice, as well as in adjuvant induced arthritis rats, two diseases with inflammatory components [11, 13]. However these EPC-based NSSL were not viable for clinical application due to rapid TMN release under storage conditions. Our new DMPC:DPPC:Chol:PEG-DSPE formulation showed an improved, slow drug-release kinetics compared to the EPC:Chol:PEG-DSPE formulation, as well as improved and prolonged therapeutic efficacy. Our results suggest that the study of NSSL–TMN for therapy of MS, and other neurodegenerative diseases involving oxidative damage, is worth pursuing.
2. We confirmed that sterically stabilized nano-liposomes (NSSL), remote loaded with the amphipathic weak acid steroid prodrug MPS by a transmembrane calcium acetate gradient, significantly ameliorates the clinical symptoms of EAE in an acute mice model [9], and also showed that the same applies to the adoptive transfer model. In this study we used a ‘‘pulse therapy” clinical treatment, in which a high dose of MPS is administrated IV for a short period of time, until the autoimmune attack is diminished. The MoA in EAE is likely related to the ability of steroids to act as immunosuppressors, to reduce edema, inhibit demyelination, and restore BBB abnormalities, which together ameliorate tissue damage. These studies, when combined with our previously described features of the optimized NSSL-MPS formulation (a high drug-to-lipid ratio, high efficiency of encapsulation, good stability during 2–8°C storage, superior pharmacokinetics and biodistribution, and a slow, zero-order drug release in vivo [9]) support further clinical development of NSSL–MPS as a potential therapeutic agent for neuro-inflammatory diseases such as MS.

## Supporting Information

S1 Checklist The ARRIVE Guidelines checklist. (DOC)

S1 Fig Comparison of the therapeutic efficacy of NSSL-MPS and EPC-based NSSL-TMN MPS in acute EAE mice model. SJL/J mice (n = 10) were treated by IV injections on days 8, 11, 14 post-immunization with: NSSL-MPS, 50 mg/kg BW (●), NSSL-TMN, 8.5mg/kg BW (■), and dextrose 5% (control) (▲).
(TIF)

S1 Table Comparison of the therapeutic efficacy of NSSL-MPS and EPC-based NSSL-TMN in acute EAE mice model. (PDF)

S1 Dataset Representing dataset extracted from raw data of EAE study. (XLSX)
