# Supplementary material for: Nano-Drugs Based on Nano Sterically Stabilized Liposomes for the Treatment of Inflammatory Neurodegenerative Diseases
Source: PLoS One. 2015 Jul 6;10(7):e0130442. doi: 10.1371/journal.pone.0130442 (PMC4492950; doi:10.1371/journal.pone.0130442)
Supplement: S1 Checklist — (DOC) [file pone.0130442.s001.doc]

**The ARRIVE Checklist**

**TITLE**

1. **Provide as accurate and concise a description of the content of the article as possible.**

Nano-drugs based on nano sterically stabilized liposomes for the treatment of inflammatory neurodegenerative diseases

**ABSTRACT**

1. **Provide an accurate summary of the background, research objectives (including details of the species or strain of animal used), key methods, principal findings, and conclusions of the study.**

The present study shows the advantages of liposome-based nano-drugs as a novel strategy for delivering active pharmaceutical ingredients for treatment of neurodegenerative diseases that involve neuroinflammation. We used the experimental autoimmune encephalomyelitis (EAE) mice model, the most commonly used animal model for multiple sclerosis (MS), an autoimmune disease characterized by severe neuroinflammation. The main challenges to overcome are the drugs’ unfavorable pharmacokinetics and biodistribution, which result in inadequate therapeutic efficacy and in drug toxicity (due to high and repeated dosage). We designed two different liposomal nano-drugs remote loaded with: (a) a “water-soluble” amphipathic weak acid glucocorticosteroid prodrug, methylprednisolone hemisuccinate (MPS) or (b) the amphipathic weak base nitroxide, Tempamine (TMN). Our results clearly show that these nano-drugs ameliorate the clinical signs and the pathology of EAE. We have further investigated the MPS nano-drug’s therapeutic efficacy and its mechanism of action in both the acute and the adoptive transfer EAE models, as well as optimizing the TMN nano-drug. The highly efficacious anti-inflammatory therapeutic feature of these two nano-medicines meets the criteria of disease-modifying drugs and supports further development and evaluation of these nano-drugs as potential therapeutic agents for diseases with an inflammatory component.

**INTRODUCTION**

**Background**

1. **Include sufficient scientific background (including relevant references to previous work) to understand the motivation and context for the study, and explain the experimental approach and rationale. Explain how and why the animal species and model being used can address the scientific objectives and, where appropriate, the study’s relevance to human biology.**

A wide range of central nervous system (CNS) disorders and especially neurodegenerative disorders are associated with neuroinflammation , as exemplified by multiple sclerosis (MS), the most common cause of neurological disability in young adults . It is characterized by the infiltration of the CNS by leukocytes, leading to substantial inflammation and damage to myelin and axons resulting in neuronal dysfunction. MS manifests itself by relapses and remissions of neurological disturbance. As a result of continuing neuroinflammation, damage to neurons and axons slowly progresses to permanent physical disability . There is no known cure for multiple sclerosis. Treatments attempt to return function after an attack, minimize new attacks, and prevent disability . The most commonly used animal model for MS is experimental autoimmune encephalomyelitis (EAE), which resembles the inflammatory acute phase of the human disease. The onset of EAE is characterized by enhanced vascular permeability of the blood-brain barrier (BBB) and the initiation of an inflammatory response . Further worsening of the disease eventually leads to severe morbidity and even death . Such diseases are especially amenable to treatment by nano-drug delivery systems since intra-lesional injection is not feasible, and the disruption of the BBB can be used as the Achilles’ heel of the disease as it enables one to achieve passive and active accumulation of nanoparticles at the disease site. We designed two liposomal nano-drugs aimed at the systemic treatment of diseases that involve inflammation including MS.

**Objectives**

1. **Clearly describe the primary and any secondary objectives of the study, or specific hypotheses being tested.**

The present study is aimed to apply, and to characterize the activity and MoA, of a novel strategy for treatment of neurodegenerative diseases that involve an inflammatory component, as exemplified by the use of an established EAE mice model.The objective of using such nano-drugs is to achieve a high drug concentration in the inflamed target tissue (the diseased CNS) while avoiding irrelevant tissues; such treatment will be much superior to an equivalent dose given as free drug. This should allow using a lower total amount of drug and frequency of administration, thereby reducing unwanted side effects. The goal of our study with the use of NSSL-MPS to treat EAE (as a model of MS) was to investigate in depth its mechanism of action (MoA).

We have previously reported that systemic administration of NSSL-TMN ameliorates the “clinical” manifestations of EAE . Further development of NSSL-TMN as a potential therapeutic agent for immune-related diseases (such as multiple sclerosis) is needed. We designed a lipid membrane with a better profile of “free volume” to fit a fast enough release rate at 37˚C but very slow drug release rate at 2-8˚C storage temperature. The features of the optimized NSSL-TMN drug were studied.

**METHODS**

**Ethical statement**

1. **Indicate the nature of the ethical review permissions, relevant licenses (e.g. Animal [Scientific Procedures] Act 1986), and national or institutional guidelines for the care and use of animals, that cover the research.**

All experiments were carried out in strict accordance with protocols approved by the Animal Ethical Care Committee of The Hebrew University of Jerusalem Medical School (MD-09-11840-5).

**Study design**

1. **For each experiment, give brief details of the study design, including:**

**a. The number of experimental and control groups.**

- Evaluation of the efficacy of BBB transporting peptides to cross the BBB and deliver drugs to the mice brains: Three healthy SJL/female mice groups (n=4) were tested: (1) Control – calcein loaded passively targeted NSSL treated group; (2) calcein loaded NSSL conjugated to short fraction of Beta-amyloid; (3) calcein loaded NSSL conjugated to short fraction of the binding site of Apo E.
- Comparison of the therapeutic efficacy of EPC:Chol:PEG-DSPE NSSL-TMN and DMPC:DPPC:Chol:PEG-DSPE NSSL-TMN: SJL/J mice (n = 10) were treated with: EPC:Chol:PEG-DSPE NSSL-TMN 8.5 mg/kg BW , DMPC:DPPC:Chol:PEG-DSPE NSSL-TMN 8.5mg/kg BW, and dextrose 5% (control).
- Comparison of the therapeutic efficacy of 50 and 10mg/kg NSSL-MPS in the acute EAE mice model: SJL mice (n=10) were treated with saline (control), 10mg/kg NSSL-MPS or 50mg/kg NSSL-MPS.
- Comparison of the therapeutic efficacy of passively targeted NSSL-MPS and actively targeted peptide-conjugated NSSL-MPS in the acute EAE mice model: SJL mice (n=12) were treated with saline (control), NSSL-MPS, Apo-E NSSL-MPS or β-amyloid NSSL-MPS.
- Comparison of the therapeutic efficacy of NSSL-MPS and free MPS in the adoptive transfer EAE mice model: SJL mice (n=10) were treated with saline (control), free MPS (50mg/kg) or NSSL-MPS (50mg/kg).
- Brain tissue characteristics of acute EAE mice using magnetic resonance imaging : SJL mice (n=10) were treated with saline (control, or NSSL-MPS (10mg/kg).

**b. Any steps taken to minimise the effects of subjective bias when allocating animals to treatment (e.g., randomisation procedure) and when assessing results (e.g., if done, describe who was blinded and when).**

- The injections were done by a technician
- The analysis was done by the researcher
- Mice were randomized before treatment

**c. The experimental unit (e.g. a single animal, group, or cage of animals).**

Group

**A time-line diagram or flow chart can be useful to illustrate how complex study designs were carried out.**

**Experimental procedures**

1. **For each experiment and each experimental group, including controls, provide precise details of all procedures carried out. For example:**

**a. How (e.g., drug formulation and dose, site and route of administration, anesthesia and analgesia used [including monitoring], surgical procedure, method of euthanasia). Provide details of any specialist equipment used, including supplier(s).**

**b. When (e.g., time of day).**

**c. Where (e.g., home cage, laboratory, water maze).**

**d. Why (e.g., rationale for choice of specific anaesthetic, route of administration, drug dose used).**

The animals were housed under standard conditions of 12-hour light/dark cycle at the Hebrew University Animal Facility and given food and water ad libitum. All experiments were carried out in strict accordance with protocols approved by the Animal Ethical Care Committee of The Hebrew University of Jerusalem Medical School (MD-09-11840-5). Daily observation, weighting and scoring of mice started on Day 7 and continued until the end of the study. During periods of active paralysis, animals were provided of moistened food on the cage floor, injected subcutaneously with saline as hydration fluid and were separated from non-affected animals. Animals were euthanized when they met the following humane endpoint criteria: loss of > 20% the initial body weight or a >10% loss compared to the previous measured body weight, mice with full clinical paralysis. Animals were sacrificed once the experiment was completed. In all cases, mice were sedated (using carbon dioxide) prior to cervical dislocation.

**Experimental animals**

1. **a. Provide details of the animals used, including species, strain, sex, developmental stage (e.g., mean or median age plus age range), and weight (e.g., mean or median weight plus weight range). b. Provide further relevant information such as the source of animals, international strain nomenclature, genetic modification status (e.g. knock-out or transgenic), genotype, health/immune status, drug- or test naıve, previous procedures, etc.**

6-7 weeks old, female SJL/J mice were obtained from Harlan Laboratories (Jerusalem, Israel).

**Housing and husbandry**

1. **Provide details of:**

**a. Housing (e.g., type of facility, e.g., specific pathogen free (SPF); type of cage or housing; bedding material; number of cage companions; tank shape and material etc. for fish).**

**b. Husbandry conditions (e.g., breeding programme, light/dark cycle, temperature, quality of water etc. for fish, type of food, access to food and water, environmental enrichment).**

**c. Welfare-related assessments and interventions that were carried out before, during, or after the experiment.**

The animals were housed under standard conditions of 12-hour light/dark cycle at the CPF unit of the Hebrew University Animal Facility and given food and water ad libitum.

Daily observation, weighting and scoring of mice started on Day 7 and continued until the end of the study. During periods of active paralysis, animals were provided of moistened food on the cage floor, injected subcutaneously with saline as hydration fluid and were separated from non-affected animals. Animals were euthanized when they met the following humane endpoint criteria: loss of > 20% the initial body weight or a >10% loss compared to the previous measured body weight, mice with full clinical paralysis. Animals were sacrificed once the experiment was completed. In all cases, mice were sedated (using carbon dioxide) prior to cervical dislocation.

**Sample size**

1. **a. Specify the total number of animals used in each experiment and the number of animals in each experimental group.**

10-12 mice per group were used in the EAE experiments in order to ensure good statistical analysis.

**b. Explain how the number of animals was decided. Provide details of any sample size calculation used.**

**c. Indicate the number of independent replications of each experiment, if relevant.**

**Allocating animals to experimental groups**

1. **Give full details of how animals were allocated to experimental groups, including randomisation or matching if done.**

**b. Describe the order in which the animals in the different experimental groups were treated and assessed.**

Before the onset of clinical signs mice were randomized. Mice were treated and assed randomly within each group.

**Experimental outcomes**

1. **Clearly define the primary and secondary experimental outcomes assessed (e.g., cell death, molecular markers, behavioural changes).**

Daily observation, weighting and scoring of mice started on Day 7 and continued until the end of the study.

**Statistical methods**

1. **a. Provide details of the statistical methods used for each analysis.**

**b. Specify the unit of analysis for each dataset (e.g. single animal, group of animals, single neuron).**

**c. Describe any methods used to assess whether the data met the assumptions of the statistical approach.**

For each group, the mean daily clinical score is given in the figures and the tables, and from the combined data the following statistical parameters are given: mean maximal score, mean day of onset, and mean burden of disease (the mean of all the scores throughout all the days of the experiment). For mean burden of disease, Student’s t-test was used to determine statistical significance at P< 0.05.

**RESULTS**

1. **Baseline data
   For each experimental group, report relevant characteristics and health status of animals (e.g., weight, microbiological status, and drug- or test-naıve) before treatment or testing (this information can often be tabulated).**

All animals analyzed were in good health.

**Numbers analysed**

1. **Report the number of animals in each group included in each analysis. Report absolute numbers (e.g. 10/20, not 50%).**

**b. If any animals or data were not included in the analysis, explain why.**

All animals were included in the analysis.

**Outcomes and estimation**

1. **Report the results for each analysis carried out, with a measure of precision (e.g., standard error or confidence interval).**

**Table 4: Comparison of the therapeutic efficacy of EPC-based NSSL-TMN and DMPC:DPPC-based NSSL-TMN in acute EAE mice model**

| **Group** | **Incidence (#dead)** | **Mean maximal score** | **Mean onset of disease (day)** | **Mean duration (days)** | **Mean burden of disease** |
| --- | --- | --- | --- | --- | --- |
| **Control** | 10/12 (0) | 2.45±0.28 | 13±0.25 | 9.2±1.1 | 0.77±0.08 |
| **EPC NSSL-TMN** | 8/11 (0) | 2.2±0.37 | 16±1.4 | 7.2±1 | 0.5±0.07 |
| **DMPC:DPPC NSSL-TMN** | 7/11 (0) | 2.1±0.32 | 17.6±1.7 | 5.5±1 | 0.26±0.05 ^a,b^ |

^a^ Significant difference from the control group P<0.0001
^b^ Significant difference from the EPC NSSL-TMN treated group P<0.001

Table 5: Comparison of the therapeutic efficacy of 50 and 10mg/kg NSSL-MPS in the acute EAE mice model

| **Group** | **Incidence (#dead)** | **Mean maximal score** | **Mean onset of disease** | **Mean duration (days)** | **Mean burden of disease** |
| --- | --- | --- | --- | --- | --- |
| **Control** | 11/11 (2) | 4.2±0.35 | 10.8±0.26 | 9.5±1.3 | 2.26±0.18 |
| **NSSL-MPS 50mg/kg** | 5/10 (0) | 1.1±0.24 ^a^ | 12.4±1.2 | 1.9±0.8 ^c^ | 0.26±0.06 ^a^ |
| **NSSL-MPS 10mg/kg** | 10/10 (0) | 1.9±0.4 ^a^ | 10.9±0.27 | 4.7±1.5 ^a^ | 0.65±0.1 ^a,b^ |

a- Significant difference from the control group P<0.0001
b- Significant difference from NSSL-50mg/kg group P<0.005
c- Significant difference from the control group P<0.05

Table 6: Comparison of the therapeutic efficacy of passively targeted NSSL-MPS and actively targeted peptide conjugated NSSL-MPS in the acute EAE mice model

| **Group** | **Incidence (#dead)** | **Mean maximal score** | **Mean onset of disease** | **Mean duration (days)** | **Mean burden of disease** |
| --- | --- | --- | --- | --- | --- |
| **Control** | 13/13 (2) | 3.9±0.35 | 10.8±0.32 | 7.5±0.38 | 2.4±0.17 |
| **NSSL-MPS** | 13/14 (0) | 2.5±0.30 ^e^ | 10.5±0.23 | 4.8±0.57 ^b^ | 1.0±0.10 ^a,c^ |
| **ApoE NSSL-MPS** | 14/14 (0) | 2.5±0.27 ^e^ | 10.6±0.20 | 4.5±0.52 ^f^ | 0.9±0.10 ^a,d^ |
| **β-amyloid NSSL-MPS** | 14/14 (1) | 3.2±0.31 | 10.5±0.17 | 6.1±0.59 | 1.7±0.14 ^b^ |

^a^ Significant difference from the control group P<0.00001
^b^ Significant difference from the control group P<0.001
^c^ Significant difference from the β-amyloid NSSL-MPS group P<0.0005
^d^ Significant difference from the β-amyloid NSSL-MPS group P<0.0001

^e^ Significant difference from the control group P<0.005
^f^ Significant difference from the control group P<0.0001

**Table 7: Comparison of the therapeutic efficacy of NSSL-MPS and free MPS in** the adoptive transfer EAE mice model

| **Group** | **Incidence (#dead)** | **Mean maximal score** | **Mean onset of disease** | **Mean duration (days)** | **Mean burden of disease** |
| --- | --- | --- | --- | --- | --- |
| **Control** | 9/9 (0) | 3.7±0.16 | 9.1±0.26 | 7.0±0.57 | 1.9±0.15 |
| **NSSL-MPS** | 6/9 (0) | 0.83±0.22 ^a^ | 11.3±0.91 | 1.7±0.57 ^c^ | 0.2±0.05 ^a^ |
| **Free MPS** | 8/8 (0) | 2.83±0.31 ^b^ | 10.1±0.35 | 2.3±0.80 ^d^ | 1.2±0.13 ^d,a^ |

^a^ Significant difference from the control group P<0.000001
^b^ Significant difference from the NSSL-MPS group P<0.0005
^c^ Significant difference from the control group P<0.00005
^d^ Significant difference from the NSSL-MPS group P<0.005

**Adverse events**

1. **a. Give details of all important adverse events in each experimental group.**

**b. Describe any modifications to the experimental protocols made to reduce adverse events.**

There were no adverse events.

**DISCUSSION**

**Interpretation/scientific implications**

1. **a. Interpret the results, taking into account the study objectives and hypotheses, current theory, and other relevant studies in the literature.**

**b. Comment on the study limitations including any potential sources of bias, any limitations of the animal model, and the imprecision associated with the results.**

**c. Describe any implications of your experimental methods or findings for the replacement, refinement, or reduction (the 3Rs) of the use of animals in research.**

- DMPC:DPPC-based NSSL-TMN demonstrated superior therapeutic efficacy compared to control group and compared to EPC-based NSSL-TMN treated group
- Treatment with NSSL-MPS showed significant therapeutic efficacy even at 5-fold lower dosage
- Both targeted formulations (ApoE- and β-amyloid-conjugated NSSL) did not show any therapeutic advantages over the passively targeted NSSL formulation, although all three formulations significantly ameliorated disease severity compared to control group.
- We tested the therapeutic efficacy of our NSSL-MPS formulation compared to the free MPS drug under stringent conditions, starting treatment at the time of first clinical signs of EAE. Although treatment with free MPS showed clear efficacy, treatment with the liposomal MPS was much more effective.
- The therapeutic effect was accompanied with changes in the brain tissue characteristics compared to control group observed by magnetic resonance imaging (MRI)

**Generalisability/translation**

1. **Comment on whether, and how, the findings of this study are likely to translate to other species or systems, including any relevance to human biology.**

Brain pathology as in the case of neuroautoimmune diseases is worsened, not only by the infiltration of activated lymphoid cells and by a high level of inflammatory cytokines, but also by the release of large amounts of free radicals . Here we address these harmful elements on two fronts with two different NSSL drugs: NSSL-MPS and NSSL-TMN.

**1)** To neutralize free radicals there is a need for a potent antioxidant with long circulation time that can penetrate the diseased brain and be active there. We have previously shown that NSSL-TMN is efficient in inhibiting EAE in mice, as well as in adjuvant induced arthritis rats, two diseases with inflammatory components . However these EPC-based NSSL were not viable for clinical application due to rapid TMN release under storage conditions. Our DMPC:DPPC:Chol:PEG-DSPE formulation showed an improved, slow drug-release rate kinetics compared to the EPC:Chol:PEG-DSPE formulation, as well as improved and prolonged therapeutic efficacy. Our results suggest that the study of NSSL–TMN for therapy of MS, and other neurodegenerative diseases involving oxidative damage, is worth pursuing.

**2)** We confirmed that sterically-stabilized nano-liposomes, remote loaded with the amphipathic weak acid steroid prodrug MPS by a transmembrane calcium acetate gradient, significantly ameliorates the clinical symptoms of EAE in an acute mice model , and also showed that the same applies to the adoptive transfer model. In this study we used a ‘‘pulse therapy’’ clinical treatment, in which a high dose of MPS is administrated IV for a short period of time, until the autoimmune attack is diminished. The MoA in EAE is likely related to the ability of steroids to act as immunosuppressors, to reduce edema, inhibit demyelination, and restore BBB abnormalities, which together ameliorate tissue damage. These studies, when combined with our previously described features of the optimized NSSL-MPS formulation (a high-drug-to-lipid ratio, high efficiency of encapsulation, good stability during 2-8˚C storage, superior pharmacokinetics and biodistribution, and a slow, zero-order drug release in vivo ) support further clinical development of NSSL–MPS as a potential therapeutic agent for neuro-inflammatory diseases such as MS.

**Funding**

1. **List all funding sources (including grant number) and the role of the funder(s) in the study.**

We thank the Barenholz Fund for its support.
