# Supplementary figures and images for: Nano-Drugs Based on Nano Sterically Stabilized Liposomes for the Treatment of Inflammatory Neurodegenerative Diseases
Source: PLoS One. 2015 Jul 6;10(7):e0130442. doi: 10.1371/journal.pone.0130442 (PMC4492950; doi:10.1371/journal.pone.0130442)

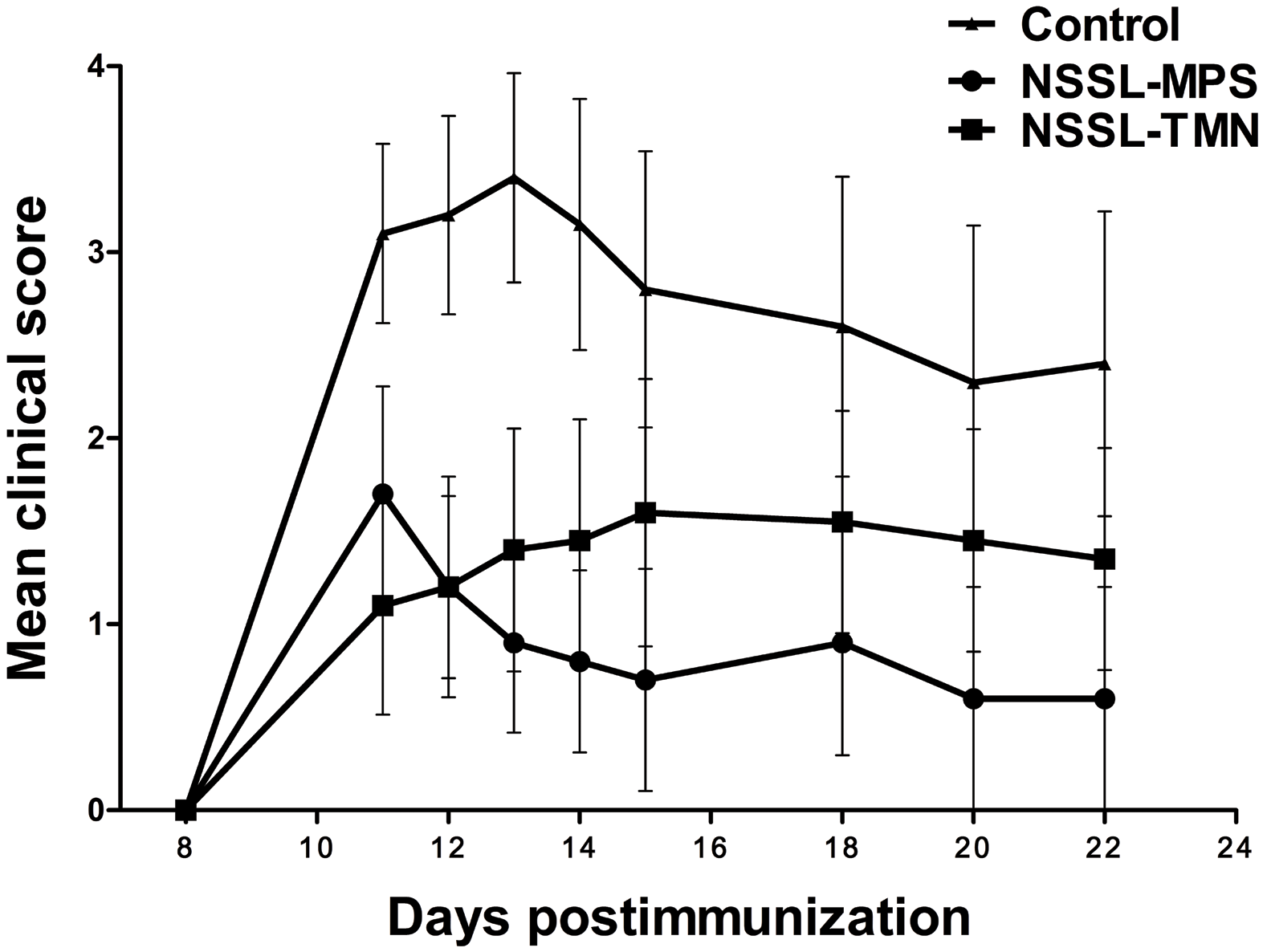

Supplement: S1 Fig — SJL/J mice (n = 10) were treated by IV injections on days 8, 11, 14 post-immunization with: NSSL-MPS, 50 mg/kg BW (●), NSSL-TMN, 8.5mg/kg BW (■), and dextrose 5% (control) (▲). (TIF) [file pone.0130442.s002.tif]
