# Supplementary material for: Nano-Drugs Based on Nano Sterically Stabilized Liposomes for the Treatment of Inflammatory Neurodegenerative Diseases
Source: PLoS One. 2015 Jul 6;10(7):e0130442. doi: 10.1371/journal.pone.0130442 (PMC4492950; doi:10.1371/journal.pone.0130442)
Supplement: S1 Table — (PDF) [file pone.0130442.s003.pdf]

S2 Table: Comparison of the therapeutic efficacy of NSSL-MPS and EPC-based NSSL-TMN in acute EAE mice model

Group	Incidence (#dead)	Mean maximal score	Mean onset of disease (day)	Mean duration (days)	Mean burden of disease
**Control**	10/10 (3)	3.7±0.517	12.1±1.1	6.4±1.13	2.54±0.223
**NSSL-MPS**	10/10 (0)	2.67±0.577	12.2±0.76	2.78±0.572	0.8±0.166 [a]
**NSSL-TMN**	10/10 (1)	2.94±0.586	14.9±1.59	5.63±1.49	1.25±0.183 [a,b]

[a] Significant difference from the control group $P<0.0001$

[b] Significant difference from the NSSL-MPS-treated group $P<0.05$
